# Plant salicylic acid signaling is inhibited by a cooperative strategy of two powdery mildew effectors

**DOI:** 10.1128/mbio.03959-24

**Published:** 2025-03-17

**Authors:** Yuhan Liu, Xiao Li, Qiguang He, Minghao Zuo, Yinjie Guo, Lijuan Liu, Jinyao Yin, Lijuan He, Xiaoli Li, Jiaxin Shan, Wenbo Liu, Chunhua Lin, Weiguo Miao

**Affiliations:** 1 Sanya Nanfan Research Institute, Key Laboratory of Green Prevention and Control of Tropical Plant Diseases and Pest (Ministry of Education)/School of Tropical Agriculture and Forestry, Hainan University, Haikou, China; 2 School of Life and Health Science, Hainan University 74629 , Haikou, China; 3 Danzhou Invasive Species Observation and Research Station of Hainan Province, Hainan University, Danzhou, China; 4 Key Laboratory of Biology and Genetic Resources of Rubber Tree, Ministry of Agriculture and Rural Affairs/State Key Laboratory Incubation Base for Cultivation & Physiology of Tropical Crops, Rubber Research Institute, Chinese Academy of Tropical Agricultural Sciences, Haikou, China; The University of British Columbia, Vancouver, British Columbia, Canada

**Keywords:** powdery mildew, salicylic acid, effector protein, plant immunity, ubiquitination

## Abstract

Powdery mildew is a global threat to crops and economically valuable plants. Salicylic acid (SA) signaling plays a significant role in plant resistance to biotrophic parasites; however, the mechanisms behind how powdery mildew fungi circumvent SA-mediated resistance remain unclear. Many phytopathogenic microbes deliver effectors into the host to sustain infection. In this study, we showed that the rubber tree powdery mildew fungus *Erysiphe quercicola* inhibits host SA biosynthesis by employing two effector proteins, EqCmu and EqPdt. These effector proteins can be delivered into plant cells to hydrolyze chorismate, the main precursor of SA, through their enzymatic activities. Notably, EqCmu and EqPdt can interact with each other, providing mutual protection against protein degradation mediated by the plant ubiquitin-proteasome system. This interaction enhances their activities in the hydrolysis of chorismate. Our study reveals a new pathogenic strategy by which two powdery mildew effector proteins cooperate to evade recognition by dampening the host immune system.

**IMPORTANCE:**

Powdery mildew fungi may develop diverse strategies to disturb salicylic acid (SA) signaling in plants, which plays an important role in activating immunity, and little is known about these strategies. Our results suggest that the *Erysiphe quercicola* effector protein EqCmu can be translocated into host cells and inhibit host SA levels during the infection stage; however, it is targeted by the plant ubiquitin–proteasome system (UPS) and ubiquitinated, which induces EqCmu degradation. To evade the UPS, EqCmu interacts with EqPdt, another *E. quercicola* effector protein, to prevent that ubiquitination. EqPdt also inhibits host SA biosynthesis through its prephenate dehydratase activity. Taken together, these two powdery mildew effector proteins cause a synergistic effect in disturbing host SA signaling. Our study also suggests that enhancing SA signaling is required for boosting immunity against powdery mildew fungus.

## INTRODUCTION

Powdery mildew fungi, a group of obligate biotrophic parasites, infect a myriad of plants, including cereals and other economically important plants. Most of these fungi exhibit a high degree of host specialization (1). Through their appressoria, they penetrate through the plant cuticle; once inside the plant tissues, they form haustoria—their feeding structures. The plasma membrane (PM) of plant cells surrounding the haustoria is known as the extrahaustorial membrane (EHM) (2). The interface between the haustoria and the EHM facilitates the nutrient acquisition of and infection by these fungi (3). Phytopathogenic microbes that form the haustoria are thought to disturb plant immunity by secreting a set of virulence factors (effectors) that are translocated through the interface or the EHM into host cells (3). Several genes encoding candidate effector proteins have been identified in powdery mildew fungi, and some have been found to play important roles in infection and host adaptation (4–7).

The gene manipulation methods applied to model fungi are unsuitable for powdery mildew fungi because they cannot grow in artificial culture media (8). Thus, to investigate the molecular basis of pathogenicity, the host-induced gene silencing (HIGS) method has been applied to evaluate effector functions (9). Using the HIGS method, host plants carrying constructs of RNA interference (RNAi) are generated via genetic transformation. However, these transformation methods have only been established for a few plant species. It has recently been reported that for some phytopathogens, including filamentous fungi and oomycetes, exogenously applied artificial RNAs, such as double-stranded RNAs (dsRNAs), can also induce targeted gene silencing, depending on the efficiency of RNA uptake by the pathogens (10, 11). The spraying-induced gene silencing (SIGS) method has shown the potential for plant disease control (10, 11). In addition, SIGS is considered appropriate for studying obligate biotrophic fungal effector functions because it can also induce targeted gene silencing in obligate biotrophic fungi, such as the cucurbit powdery mildew fungus *Podosphaera xanthii*, the *Arabidopsis* powdery mildew fungus *Golovinomyces orontii,* the rubber tree powdery mildew fungus *Erysiphe quercicola*, and the Asian soybean rust fungus *Phakopsora pachyrhizi* (12–16).

To prevent infection by pathogens, plants employ immune receptors localized on the cell surface and in the cytosol to detect pathogen-associated molecular patterns and avirulence effectors, leading to the activation of immune signaling pathways, such as the salicylic acid (SA) signaling pathway (17–19). SA is a phytohormone derived from chorismate, and SA levels in plants become elevated under biotic stress (20, 21). The conversion of chorismate into isochorismate, which is facilitated by isochorismate synthases (ICSs), is a critical step in SA biosynthesis (20, 21). Isochorismate and glutamic acid form isochorismate-9-glutamate, which decomposes into SA (20, 21). In the plant shikimate pathway, chorismate is also converted into aromatic amino acids such as phenylalanine by chorismate mutases (CMUs) and prephenate dehydratases (PDTs) (20). CMUs catalyze chorismate into prephenate, and then prephenate is converted into phenylpyruvate by PDTs for phenylalanine formation (22–24).

To invade a host plant, the corn smut fungus *Ustilago maydis* and plant parasitic nematodes have developed a strategy to attenuate plant SA biosynthesis by co-opting the plant shikimate pathway (25). These pathogens secrete CMU effector proteins with enzymatic activity to promote the conversion of chorismate into prephenate in the host plant, thereby decreasing the amount of chorismate converted into isochorismate for SA biosynthesis (25–28). Therefore, secreted CMUs are indispensable for full pathogenesis.

The powdery mildew fungus *E. quercicola* (also known as *Oidium heveae*) infects rubber trees (*Hevea brasiliensis* Muell), the primary source of natural rubber, causing an approximately 40% reduction in annual natural rubber production (29, 30). The *E. quercicola* genome has been sequenced, and the candidate effector genes of this fungus have been annotated (6). The putatively secreted CMU protein of *E. quercicola*, EqCmu, is hypothesized to act as a virulence effector and to suppress plant SA signaling, because EqCmu was found to display CMU activity in an enzyme activity assay (31). Heterogeneous EqCmu expression decreased SA levels in the model plant *Nicotiana benthamiana* (31). However, the specific roles of EqCmu in the host–*E*. *quercicola* interaction were less studied.

In this study, we further investigated the characteristics of EqCmu via SIGS and SA measurements as well as other approaches. Surprisingly, we found that EqCmu could be targeted by the host ubiquitin–proteasome system (UPS). The UPS is responsible for substrate protein modification and degradation in living cells, and it plays an important role in plant immunity (32–34). The ubiquitination process is catalyzed by three classes of core enzymes, including ubiquitin-activating enzymes (E1s), ubiquitin-conjugating enzymes (E2s), and ubiquitin ligases (E3s). During the process, ubiquitin, a conserved small protein, is activated by E1s and conjugated to the target by E2s and E3s (32–34). The resultant ubiquitin-conjugated protein is then recognized and degraded by the 26S proteasome (32, 33). Although our results reveal that EqCmu is an important effector protein for inhibiting SA biosynthesis in the host, the plant UPS reduced EqCmu activity to enhance defense response, whereas EqCmu could interact and function with EqPdt, an effector protein with PDT activity, to escape from recognition by the UPS. These findings contribute to a better understanding of the specific and sophisticated mechanisms adopted by powdery mildew effectors.

## RESULTS

### SA contributes to *H. brasiliensis* resistance to the powdery mildew fungus

**Fig 1:**
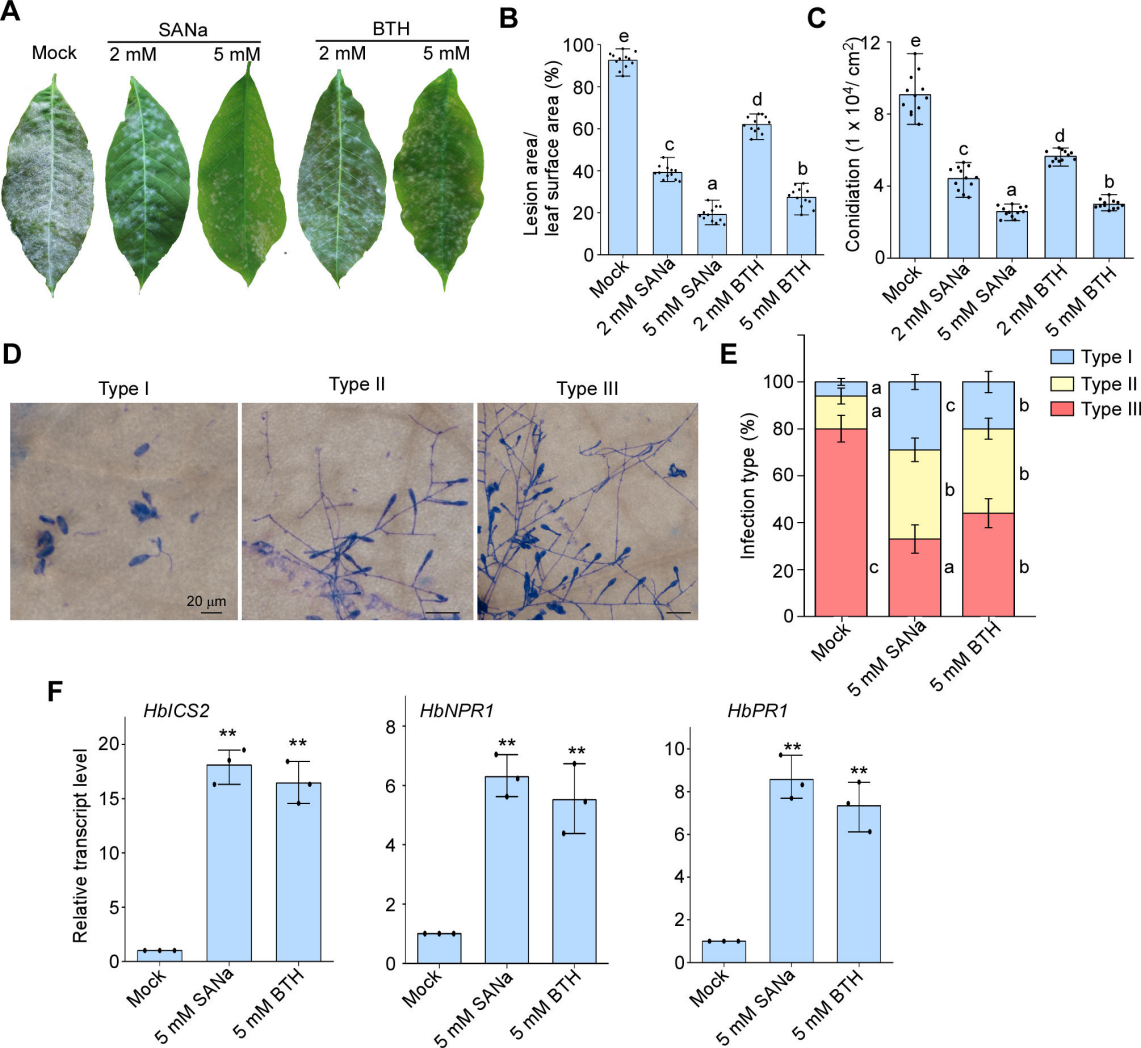
Exogenous application of salicylic acid (SA) analogs to *H. brasiliensis* induced resistance to *E. quercicola*. (**A**) Representative photographs taken at 7 dpi showing that SANa or BTH treatment to *H. brasiliensis* leaves inhibited *E. quercicola* growth. SANa or BTH solutions (2 and 5 mM) were sprayed onto *H. brasiliensis* leaves, followed by inoculation with *E. quercicola* conidium suspension (1 × 10^5^ spores/mL). (**B**) The percentage of lesion area in total area per *H. brasiliensis* leaf. Bar chart shows the means and standard deviation (SD; *n* = 12 leaves from three independent experiments). In panels B, C, and E, one-way analysis of variance (ANOVA) with Tukey’s test was used to analyze statistical difference indicated by different letters (*P* < 0.01). (**C**) Conidia in infected *H. brasiliensis* leaves were quantified. Bar chart shows the means and SD (*n* = 12 leaves from three independent experiments). (**D**) Infection types of *E. quercicola* at 48 hpi. Type I, conidia with no penetration; type II, the elongating hyphae shorter than 150 µm; type III, the elongating hyphae longer than 150 µm. (**E**) Quantification of infection types. Bar chart showing the means and SD (*n* = 300 germinated conidia from three independent experiments). (**F**) Relative transcript levels of *HbICS2*, *HbNPR1*, and *HbPR1* were determined by conducting qRT-PCR. SANa or BTH solutions were applied to *H. brasiliensis* leaves for 24 h, and the total RNA collected from these leaves was analyzed through qRT-PCR. *H. brasiliensis Actin* gene was used as an internal control. Bar chart showing the mean and SD with three biological replicates (*n* = 3 for each replicate). The mock was compared with each of the other samples using a two-tailed Student’s *t*-test. Significant differences (*P* < 0.01) are indicated by asterisks.

Previous studies have reported that SA regulates the resistance of grapevines and Arabidopsis to powdery mildew (35, 36). To investigate whether SA plays a role in regulating *H. brasiliensis* resistance to *E. quercicola*, SA agonists, including sodium salicylate (SANa) and benzothiadiazole (BTH) (37, 38), were used to treat *H. brasiliensis* leaves prior to inoculation with *E. quercicola*. Compared with the H_2_O treatment (mock control), the SANa and BTH treatments significantly reduced fungal growth by inhibiting hyphal expansion and conidiation (Fig. 1A through E). The inhibitory effect of 5 mM SANa or BTH on the pathogen was the most evident, thus these effects of SANa and BTH treatments were concentration-dependent. Additionally, quantitative reverse transcription PCR (qRT-PCR) analysis revealed that SANa and BTH treatments without *E. quercicola* inoculation increased the transcription of *H. brasiliensis ICS2* (the only *ICS* gene present in *H. brasiliensis* and homologous to *AtICS2*), *NPR1,* and *PR1*, which are marker genes of the SA-dependent immunity pathway (Fig. 1F) (39–41). Therefore, SA regulates plant immunity against *E. quercicola*.

### Screening for potential *E*. *quercicola* effectors associated with host SA-mediated immunity

**Fig 2:**
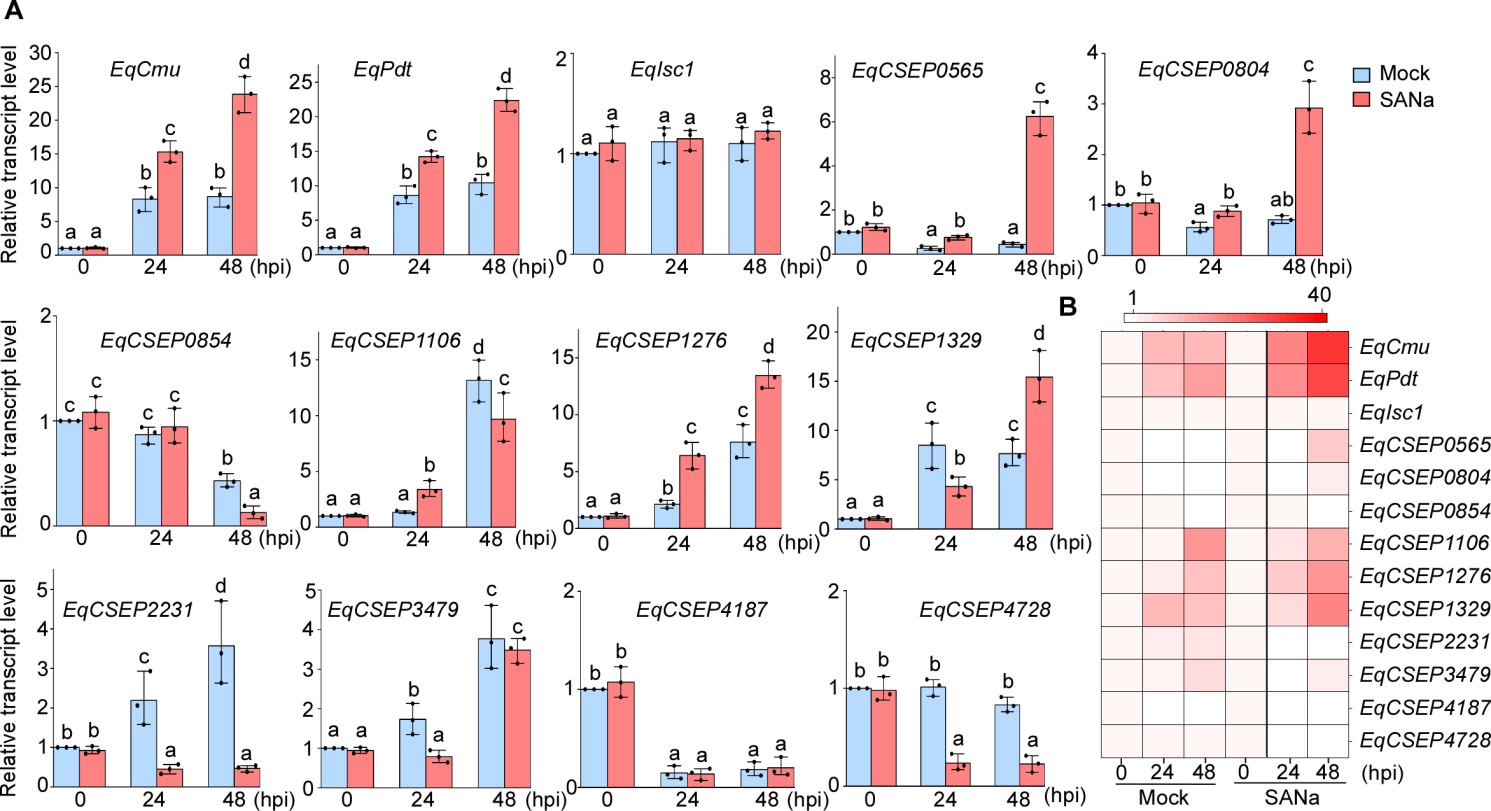
Relative transcript analysis of candidate effectors in *E. quercicola*-inoculated *H. brasiliensis* of induced SA-mediated immunity. (**A**) Relative transcript levels of candidate effector genes in *E. quercicola*-inoculated mock (H_2_O) or SANa (5 mM) treated *H. brasiliensis* leaves. Total RNA of *E. quercicola* was collected at 0, 24, and 48 hpi, and the transcript levels of candidate effectors were detected through qRT-PCR. *E. quercicola EF-1α* gene was used as an internal control. Bar charts show the mean and SD with three biological replicates (*n* = 3 for each replicate). One-way ANOVA with Tukey’s test was used to analyze statistical differences indicated by different letters (*P* < 0.01). (**B**) Heatmap showing transcript levels of candidate effector genes in panel **A**. The fold changes of the relative transcripts are indicated by different colors.

To understand how *E. quercicola* overcomes host SA-mediated immunity, we screened for potential effector proteins by analyzing their transcription upon the activation of SA-mediated immunity. Three putative secreted enzymes, including *E. quercicola* CMU, PDT, and ISC (EqCmu [31], EqPdt, and EqIsc1), were selected for the screening assay. Although these proteins lack signal peptides, they were predicted to be unconventionally secreted proteins (Table S1). Previous studies have shown that the CMU and ISC effector proteins from other filamentous pathogens target the isochorismate (IC) pathway for SA biosynthesis (25, 42). We also hypothesized that EqPdt may function with EqCmu in reducing chorismite content for SA biosynthesis in host cells. Genomic analysis has revealed that *E. quercicola* produces 133 candidate-secreted effector proteins (CSEPs), which is within the expected range for a dicot-infecting powdery mildew species (6, 43). We additionally selected 10 CSEPs as candidates for the screening (Table S2). In contrast to unconventionally secreted proteins, these CSEPs contain N-terminal signal peptides and have no homologs in non-powdery mildew species (6). Moreover, most of them do not possess conserved domains identifiable in the Pfam database (Table S2). In this screening assay, *E. quercicola* was allowed to infect *H. brasiliensis* leaves treated with H_2_O or SANa, and the transcript levels of these candidate genes at haustorium formation (24 hours post-inoculation [hpi]) and secondary penetration stages (48 hpi) (31) were determined by qRT-PCR. The results indicated that six candidate genes were upregulated following either H_2_O or SANa treatment. Notably, the upregulation levels of EqCmu and EqPdt after SANa treatment were significantly greater than those of the other genes (Fig. 2). Therefore, here, we selected EqCmu and EqPdt for functional characterization.

### EqCmu and EqPdt are important for infection

Recently, SIGS has been employed to silence the CYP5 gene in *E. quercicola* (16). To investigate effector functions, we also assessed the efficiency of SIGS in *E. quercicola*. We measured the ability of *E. quercicola* to absorb exogenous double-stranded RNA (dsRNA). A 400 nt dsRNA labeled with fluorescein was generated using the *GFP* gene as a template and was subsequently used to treat *E. quercicola* colonies. After treatment, fluorescein signals were detected on the surfaces of conidia and hyphal cells at 6 h but inside these cells at 24 h (Fig. S1A). Meanwhile, a 200 nt dsRNA homologous to *E. quercicola Tub2* (*EqTub2*) gene was generated and used to treat the colonies, which reduced the transcript level of *EqTub2* by 72.9% ± 13.1% (Fig. S1B) and inhibited fungal growth significantly (Fig. S1C and D). These results suggest that SIGS can be applied to investigate this fungus, consistent with the findings of a previous study (16).

**Fig 3:**
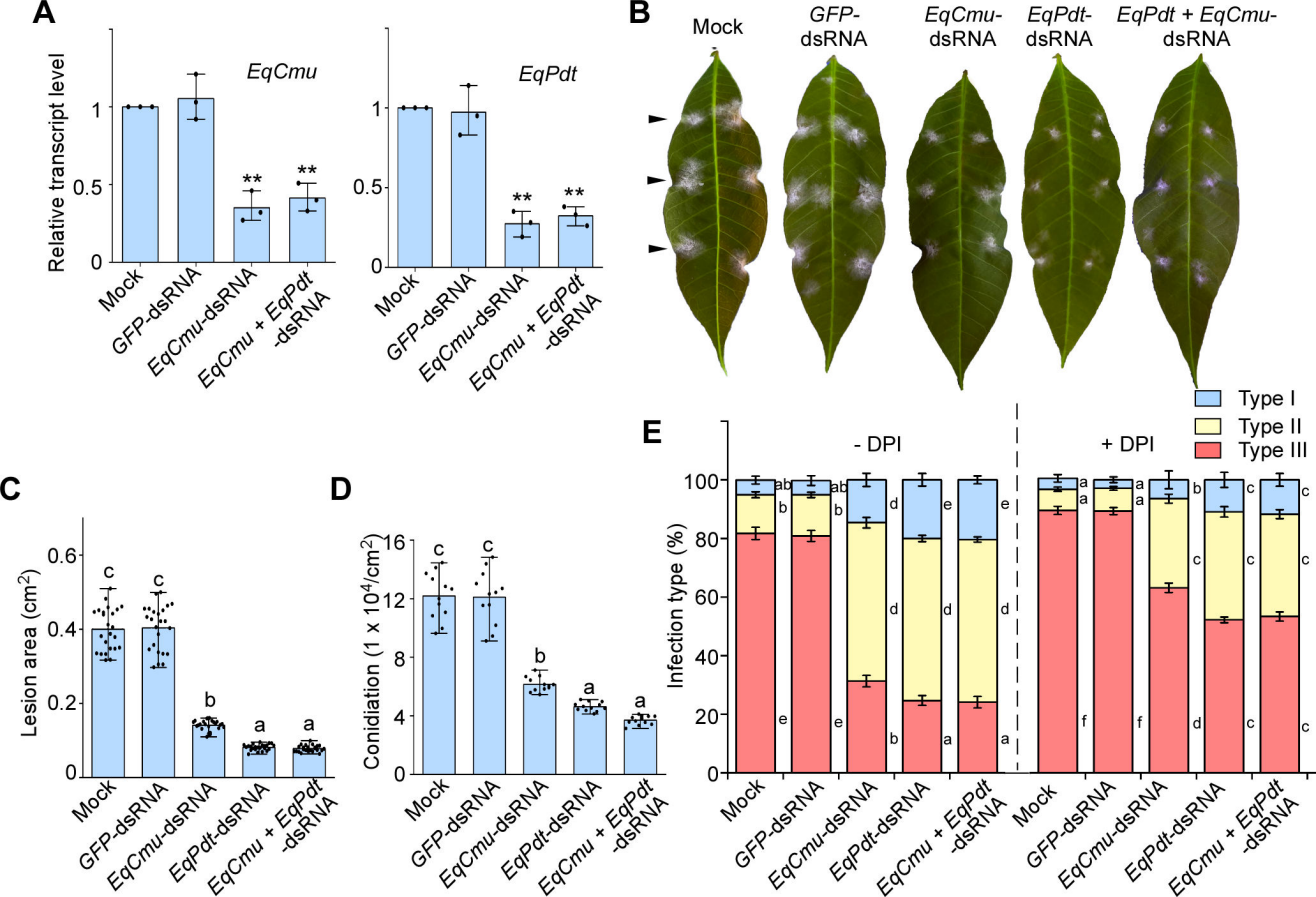
Gene silencing of *EqCmu* and *EqPdt* by dsRNA treatment reduced *E. quercicola* pathogenicity. (**A**) Relative transcript levels of *EqCmu* and *EqPdt* in *E. quercicola*. Total RNA of the fungus was collected at 5 days post-inoculation (dpi). Bar chart shows the means and SD with three biological replicates (*n* = 3 for each replicate). *EqCmu + EqPdt*-dsRNA, application of both *EqCmu*-dsRNA and *EqPdt*-dsRNA. The mock was compared with each of the other samples using a two-tailed Student’s *t*-test. Significant differences (*P* < 0.01) are indicated by asterisks. (**B**) Gene silencing of *EqCmu* and *EqPdt* reduced pathogenicity. The photographs were taken at 5 dpi. Arrows indicate inoculation sites. (**C**) Quantification of the lesion areas on *H. brasiliensis* leaves. Bar chart shows the means and SD (*n* = 24 from three independent experiments). In panels C, D, and E, one-way ANOVA with Tukey’s test was used to analyze statistical difference indicated by different letters (*P* < 0.01). (**D**) Quantification of conidia produced by the *E. quercicola* strains. Bar chart shows the means and SD (*n* = 12 from three independent experiments). (**E**) Quantification of the infection type of *E. quercicola* in leaves with or without DPI (10 µM) treatment at 48 hpi. Type I, conidia with no penetration; type II, the elongating hyphae were shorter than 150 µm; type III, the elongating hyphae were longer than 150 µm. Bar chart shows the means and SD (*n* = 300 geminated conidia from three independent experiments).

To induce the gene silencing of *EqCmu*, *EqPdt*, or both, two dsRNAs homologous to *EqCmu* and *EqPdt* (400 and 491 nt, respectively) were generated and used to treat *E. quercicola*. After 5 days, the transcript levels of *EqCmu* and *EqPdt* decreased by approximately 70% compared with those in strains treated with mock (H_2_O) and *GFP*-dsRNA, which served as negative controls (Fig. 3A). The *EqCmu*- and *EqPdt*-silenced strains, as well as the strain with both genes silenced, exhibited reduced infection abilities (Fig. 3B and C), as evidenced by inhibited hyphal growth and conidiation (Fig. 3D and E). Co-silencing of *EqCmu* and *EqPdt* had the most significant inhibitory effect on infection and fungal growth. Moreover, upon pretreatment of *H. brasiliensis* leaves with the NADPH oxidase inhibitor diphenylene iodonium (DPI), which is supposed to disturb plant basal immunity (44, 45), these silenced strains significantly restored their infection ability and hyphal growth (Fig. 3E; Fig. S2); therefore, EqCmu and EqPdt are required for suppressing host plant immunity. However, the pathogenicity of these gene-silenced strains was not fully restored in the presence of DPI, likely due to the roles of EqCmu and EqPdt in aromatic amino acid biosynthesis, which may also contribute to fungal growth.

### EqCmu and EqPdt inhibit the SA signaling pathway

**Fig 4:**
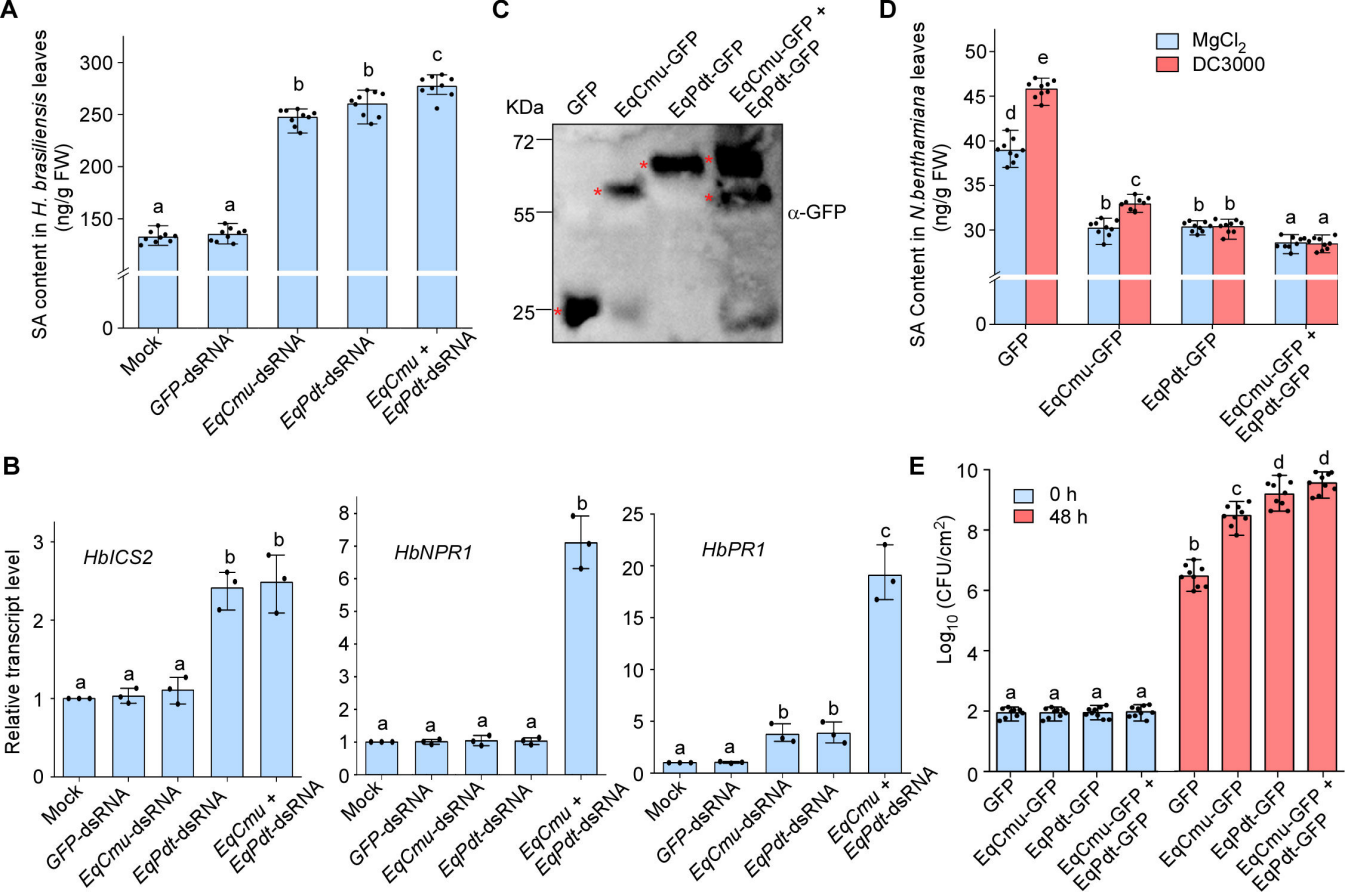
EqCmu and EqPdt inhibited plant SA synthesis. (**A**) SA content in infected *H. brasiliensis* leaf tissues was measured using LC/MS-MS at 3 dpi (*n* = 9 leaf discs from three independent experiments). Bar chart shows the means and SD. In panels **A, B, D, and E**, one-way ANOVA with Tukey’s test was used to analyze statistical difference indicated by different letters (*P* < 0.01). (**B**) Relative transcript levels of *HbICS2*, *HbNPR1,* and *HbPR1* were determined by qRT-PCR analysis. Total RNA samples of *H. brasiliensis* leaves inoculated with *E. quercicola* strains were collected at 3 dpi. *H. brasiliensis Actin* gene was used as an internal control. Bar chart shows the means and SD with three biological replicates (*n* = 3 for each replicate). (**C**) Western blotting of GFP, EqCmu-, and EqPdt-GFP in the total extracts from transgenic *N. benthamiana* leaves. The bands of these proteins were labeled by asterisks. (**D**) SA content in *N. benthamiana* leaf tissues was measured using LC/MS-MS. Leaf discs were collected for LC/MS-MS analysis at 48 h after DC3000 inoculation or infiltration with MgCl_2_ solution. Bar chart shows the means and SD (*n* = 9 leaf discs from three independent experiments). (**E**) Bacterial growth in *N. benthamiana* leaf tissues was measured. Leaf discs (1 cm^2^) were excised from the infected area and homogenized in 10 mM MgCl_2_ after DC3000 inoculation at 0 and 48 h. Bar chart shows the means and SD (*n* = 9 leaf discs from three independent experiments).

To investigate how EqCmu and EqPdt affect host SA signaling, we measured the SA content in *H. brasiliensis* leaves inoculated with those gene-silenced, as well as with H_2_O (mock control) and *GFP*-dsRNA-treated strains, using high-performance liquid chromatography (HPLC) (Fig. S3A). The results indicated that the SA content in leaves inoculated with *EqCmu*- and *EqPdt*-silenced strains was much higher than that in the controls (mock and *GFP*-dsRNA-treated strains) (Fig. 4A). Notably, the maximum SA content was observed in leaves inoculated with the strain in which both *EqCmu* and *EqPdt* were silenced (Fig. 4A). We performed qRT-PCR to determine the transcript levels of plant SA signaling marker genes. The transcript levels of *HbICS2*, *HbNPR1*, and *HbPR1* were elevated in leaves inoculated with the silenced strains compared with the controls (Fig. 4B). The highest transcript levels of these genes were induced with the strain in which both *EqCmu* and *EqPdt* were silenced. Additionally, we investigated whether EqCmu and EqPdt directly affected plant SA biosynthesis using the *N. benthamiana–Pseudomonas syringae* interaction system (42). We conducted *Agrobacterium*-mediated transformation to transiently express EqCmu-GFP and EqPdt-GFP in *N. benthamiana* leaves (Fig. 4C) and then inoculated the leaves with the bacterial pathogen *P. syringae* DC3000 or MgCl_2_ (the buffer used to suspend DC3000). The SA content in *N. benthamiana* leaves was measured 48 h after inoculation with DC3000. The results showed that the SA content in the leaves expressing GFP was higher than that in the leaves expressing EqCmu-GFP, EqPdt-GFP, or both, before and after DC3000 inoculation (Fig. 4D). Consistent with the role of SA in regulating resistance to DC3000 (46), an increase in DC3000 growth was observed in the leaves expressing EqCmu-GFP, EqPdt-GFP, or both (Fig. 4E). Therefore, both EqCmu and EqPdt can directly affect plant SA biosynthesis.

### EqCmu and EqPdt can be translocated into host cells following secretion

**Fig 5:**
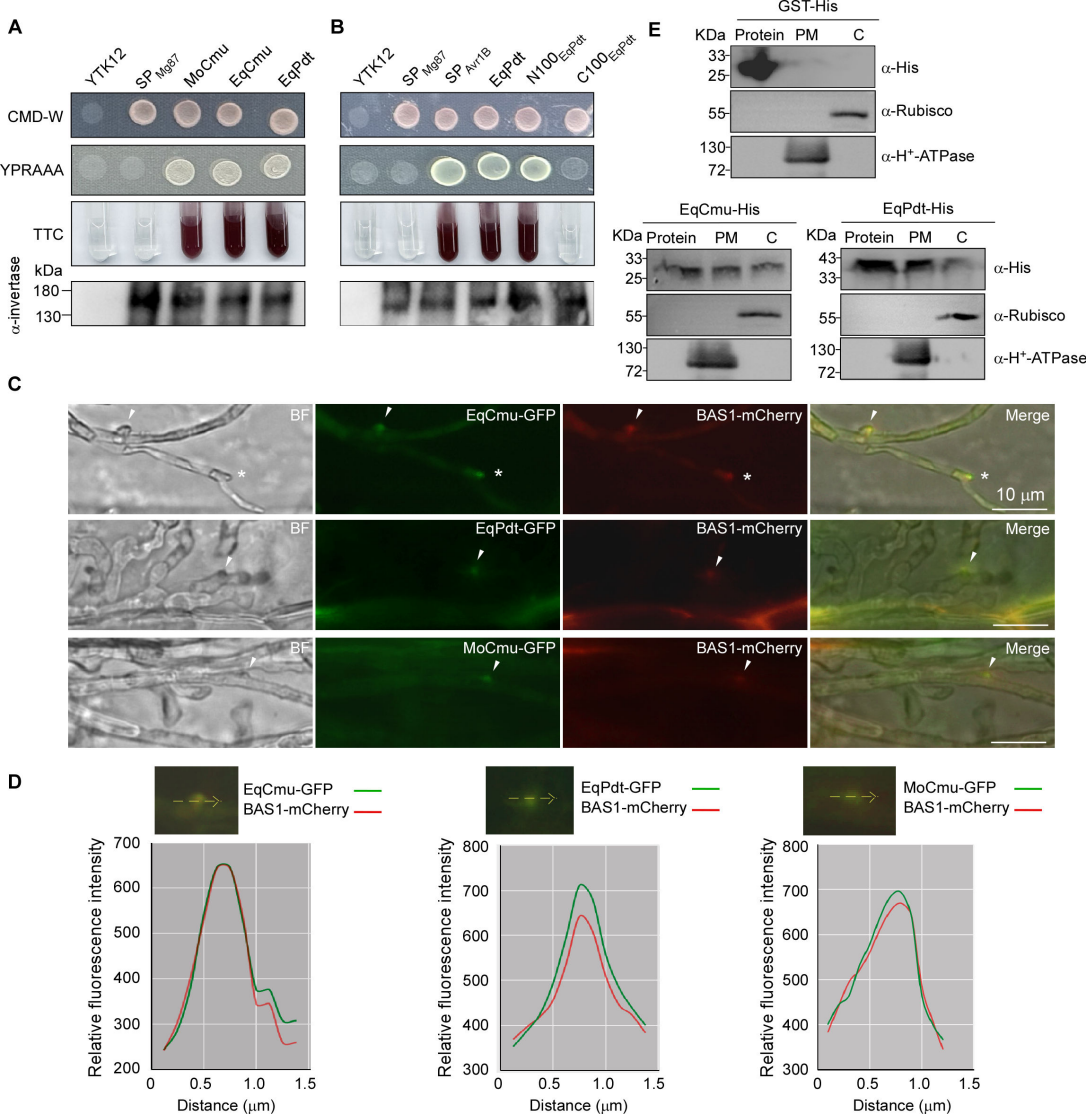
EqCmu and EqPdt could be translocated into host cells. (**A and B**) Yeast invertase secretion assay for EqCmu and EqPdt. The encoding sequences were fused into the pSUC2 vector, and the resulting vectors were introduced into the yeast YTK12 strain. The full-length versions of EqCmu, EqPdt, and MoCmu were examined in panel **A**. The N-terminal or C-terminal peptides of EqPdt (N100_EqPdt_ and C100_EqPdt_), which contain 100 amino acids, were examined in panel **B**. The signal peptides of Avr1b and Mg87 (47) were used as positive and negative controls, respectively. (**C**) EqCmu-GFP, EqPdt-GFP, and MoCmu-GFP were secreted into BICs labeled by BAS1-mCherry in barley epidermal cells infected by *M. oryzae*. For each strain, 20 infected sites with BICs were recorded at 30 hpi. The white arrows indicate BICs. Bars, 10 µm. (**D**) Fluorescence intensity linescans for GFP and mCherry along the path of a yellow arrow in confocal sections including a BIC. (**E**) Western blotting of GST-, EqCmu-, and EqPdt-His by anti-His primary antibodies and HRP-conjugated secondary antibody. Protein, purified fraction derived from *E. coli*; PM, the plasma membrane fraction of protoplasts; C, the cytosolic fraction of protoplasts.

The secretory activity of EqCmu has been validated through a yeast invertase secretion assay (47). Here, we conducted the assay again with another CMU homolog (MoCmu) derived from the rice blast fungus *Magnaporthe oryzae*. Similar to EqCmu, MoCmu is predicted to be an unconventionally secreted protein (Table S1). In this assay, both EqCmu and MoCmu displayed the ability to confer invertase secretion. These two proteins enabled the yeast invertase to grow on yeast extract peptone raffinose acetate acetamide aga（YPRAAA) medium and to catalyze the reduction of 2,3,5-triphenyltetrazolium chloride (TTC) to red-colored compounds instead of the negative strain Mg87-YTK12 (Fig. 5A) (48). We also examined the secretory activity of EqPdt by this method and found that EqPdt, or its first 100 N-terminal amino acids, could confer invertase secretion (Fig. 5B). The delivery of effector into the special plant-fungal interfaces including biotrophic interfacial complexes (BICs) and extra-invasive hyphal membranes by *M. oryzae* during infection can be monitored (49). We generated *M. oryzae* strains co-expressing a GFP-fused CMU or PDT with BAS1-mCherry. BAS1 is an effector from *M. oryzae* and preferentially accumulates in BICs (50, 51). The native *MoCmu* promoter was used to control the expression of MoCmu-, EqCmu-, and EqPdt-GFP, while the native *BAS1* promoter was used to control BAS1-mCherry expression. The *M. oryzae* transformants were allowed to infect barley leaves. In the infection sites where BICs formed, we detected the accumulation of these GFP-fused proteins and BAS1-mCherry in BICs (Fig. 5C and D). Taken together, our results suggest that EqCmu and EqPdt are secreted proteins.

We investigated effector translocation during *H. brasiliensis* leaf infection. Given that the low-abundance secreted proteins are probably insufficient for our examination, we supplemented the pathogen with additional *in vitro*-produced effector proteins during infection. *In vitro*-purified His-tagged EqCmu and EqPdt proteins (EqCmu- and EqPdt-His) were exogenously applied to *E. quercicola* strain that had been grown for 4 days in *H. brasiliensi*s leaves. Immunofluorescence analysis showed that these two proteins, along with His-tagged glutathione-S-transferase (GST-His) used as a control, accumulated on the surface of hyphae and infiltrated into the EHM at 12 hours after protein application (Fig. S4). The exogenously supplied proteins present at the plant-fungal interface mimicked effectors secreted by *E. quercicola*. We used these proteins to investigate whether EqCmu and EqPdt were translocated into leaf cells. Protoplasts (Fig. S5) were isolated from the infected leaf tissues that had been supplied with exogenous proteins, and the PM and cytosolic fractions of these protoplasts were extracted, followed by Western blot analysis (Fig. 5E). The analyses of the PM H^+^-ATPase and cytosolic Rubisco suggest that these two fractions did not mix with each other (Fig. 5E). We detected EqCmu- and EqPdt-His, but not GST-His, in both fractions. Therefore, in host leaf tissues, EqCmu and EqPdt likely adhere to the plant PM followed by the entry into the cytosol.

### EqCmu and EqPdt catalyze the conversion of chorismate to phenylalanine

The CMU activity and active enzyme sites of EqCmu have been characterized (31). In this study, EqPdt and several reported PDTs were aligned using the BioEdit software (7.2), revealing similarities between EqPdt and the reported PDTs (Fig. S6A). The conserved PDT and aspartate kinase, chorismate mutase, and TyrA (ACT) domains were predicted based on the PFAM database (52) (Fig. S6B). To determine the PDT activity of EqPdt in catalyzing the conversion of prephenate into phenylpyruvate for phenylalanine formation, we generated a *Saccharomyces cerevisiae mutant strain* Δ*ScPha2*, in which the PDT-encoding gene *ScPha2* (53) was deleted through homologous recombination. This mutant was subsequently transformed with *HA*-tagged *EqPdt* and *ScPha2*, respectively. Δ*ScPha2* could not grow in the Sabouraud dextrose (SD) medium lacking phenylalanine, while growth was restored upon complementation with EqPdt- and ScPha2-HA (Fig. S7). These results indicate that EqPdt can function as an enzyme in amino acid metabolism within fungal cells prior to secretion.

**Fig 6:**
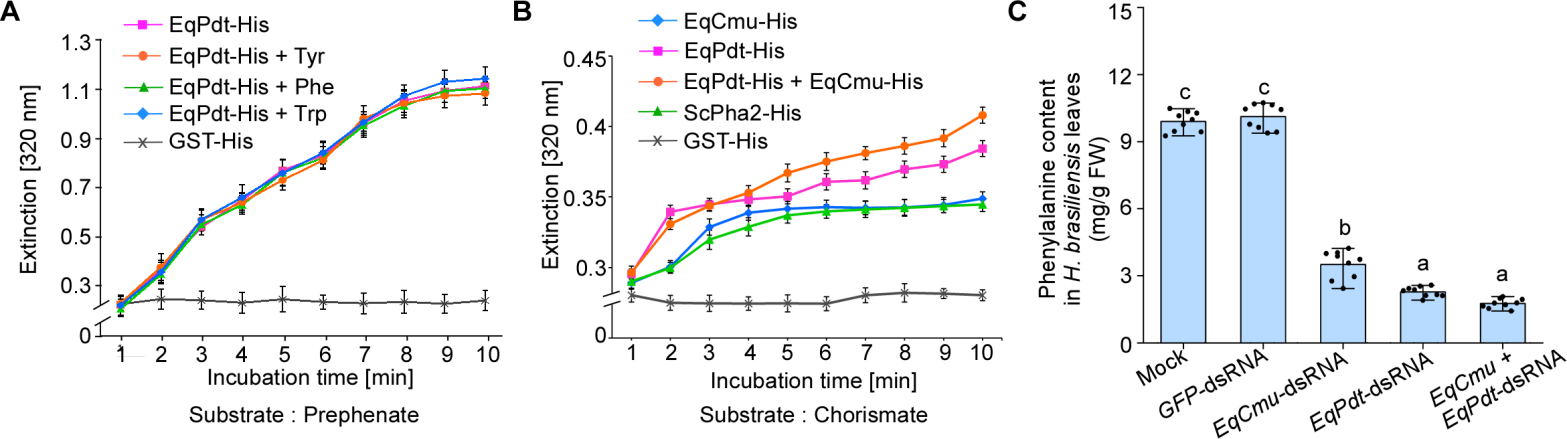
EqCmu and EqPdt displayed chorismate mutase and prephenate dehydratase activities, respectively. (**A**) EqPdt enzymatic activity was determined by measuring the absorbance at 320 nm. The purified EqPdt- and GST-His (5 µg of each protein) were incubated with prephenate, followed by the measurement of phenylpyruvate absorbance (320 nm) at the indicated time points. EqPdt exhibited PDT activity and was not involved in feedback regulation by tyrosine (Tyr), phenylalanine (Phe), or tryptophan (Trp). Solid lines and error bars represent the means and SD (*n* = 9 from three independent experiments). (**B**) Enzymatic activities of EqCmu and EqPdt were determined *in vitro*. The purified EqCmu-, EqPdt-, or ScPha2-His proteins (5 µg of each protein) were incubated with chorismate, following the measurement of phenylpyruvate absorbance (320 nm) at the indicated time points. Solid lines and error bars represent the means and SD (*n* = 9 from three independent experiments). (**C**) Phenylalanine content in *H. brasiliensis* tissues inoculated with H_2_O- and dsRNA-treated strains. One-way ANOVA with Tukey’s test was used to analyze statistical difference indicated by different letters (*P* < 0.01) (*n* = 9 from three independent experiments).

We conducted an *in vitro* enzymatic activity assay and found that EqPdt-His, but not GST-His, catalyzed the conversion of the substrate prephenate to phenylpyruvate (Fig. 6A; Fig. S8). By aligning the EqPdt sequence with other characterized PDT sequences, we identified two conserved motifs: the TRF motif in the PDT domain and the NSRP motif in the ACT domain (Fig. S9A). We mutated Thr in the TRF motif to Ala (T223A) and Asn in the NSRP motif to Glu (N275E), respectively, and then examined the enzymatic activities of these two mutant forms of EqPdt *in vitro*. The T223A mutation completely abolished the activity, while the N275E mutation reduced the activity by ~50% (Fig. S9B). Additionally, EqPdt^T223A^-GFP was expressed in *N. benthamiana* and lost its ability to suppress SA accumulation induced by DC3000 (Fig. S9C). Therefore, both the TRF and NSRP motifs are essential for the PDT activity. Meanwhile, in the presence of 0.5 mM phenylalanine, tyrosine, and tryptophan, none of these amino acids significantly altered the enzymatic activity, suggesting that EqPdt is not deactivated by aromatic amino acids (Fig. 6A). In contrast, the activities of some bacterial PDTs can be inhibited by aromatic amino acids (54, 55), as these PDTs possess the ESRP region instead of NSRP (55). ESRP is responsible for binding with aromatic amino acids (53). Studies on *Escherichia coli* PDT demonstrate that changes in ESRP result in desensitization of PDT to inhibition by aromatic amino acids (55).

Next, we investigated whether EqCmu and EqPdt functioned synergistically to hydrolyze chorismate *in vitro*. The results showed that the combination of EqCmu- and EqPdt-His catalyzed the conversion of chorismate into phenylpyruvate more effectively than EqCmu-His alone (Fig. 6B). Notably, when only chorismate was used as the substrate, EqPdt- or ScPha2-His also catalyzed the production of phenylpyruvate (Fig. 6B), likely due to the functional similarities between CMUs and PDTs.

To understand whether EqCmu and EqPdt also exert their effects through enzymatic activities in the host, *H. brasiliensis* leaves were inoculated with *E. quercicola* strains, followed by the measurement of phenylalanine content in these leaves. The results showed that the phenylalanine content in leaves inoculated with *EqCmu-* and *EqPdt*-silenced strain, as well as the strain with both genes silenced, significantly decreased compared with that of the controls (Fig. 6C; Fig. S3B). Taken together, EqCmu and EqPdt may function synergistically through their enzymatic activities.

### EqCmu can interact with EqPdt

**Fig 7:**
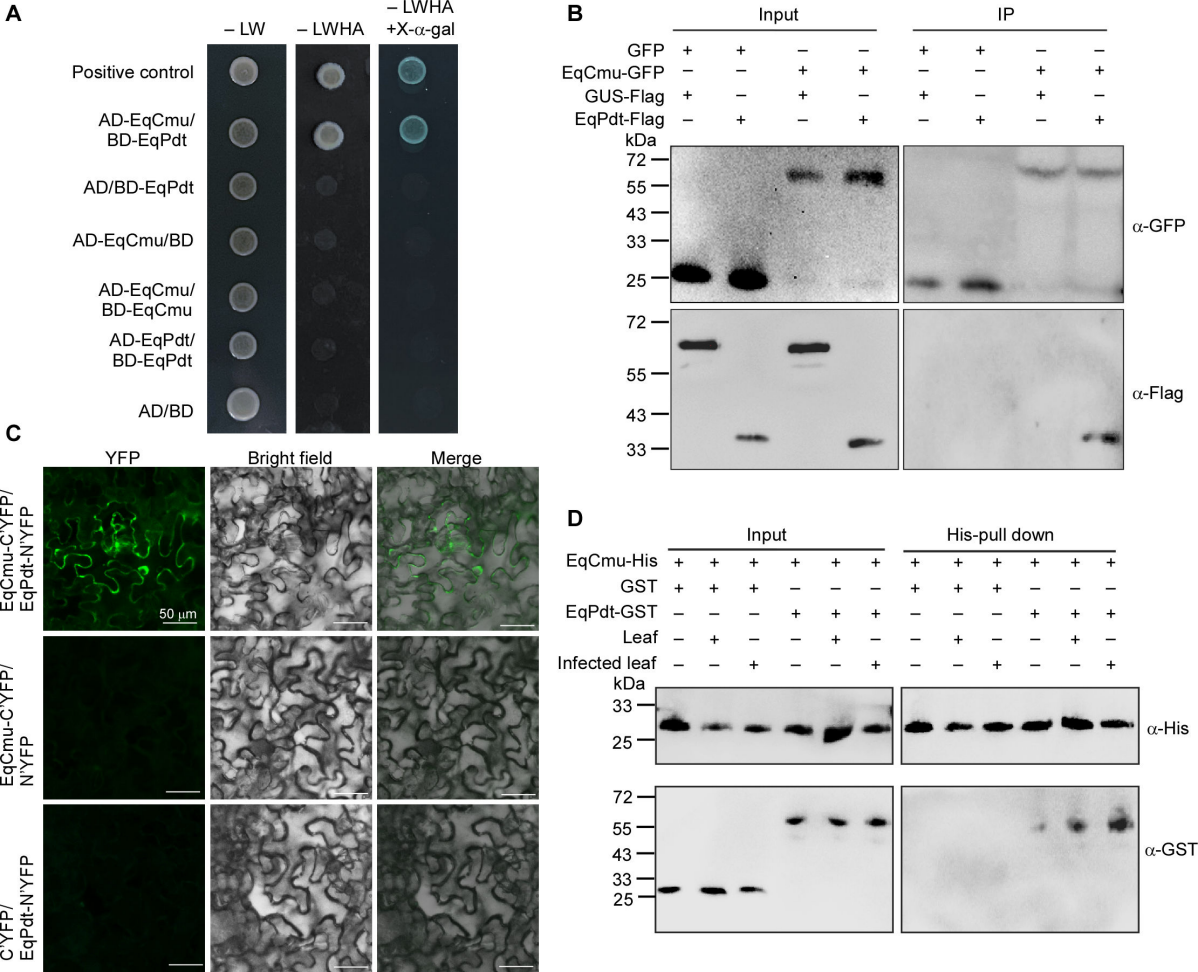
The interaction between EqCmu and EqPdt. (**A**) Yeast two-hybrid to test the interaction between EqCmu and EqPdt. The yeast strains that were transformed with recombinant AD and BD constructs were assayed for growth on SD medium lacking leucine and tryptophan (SD-LW) and on SD medium lacking leucine, tryptophan, histidine, and adenine (SD-LWHA) with X-α-gal addition. The combination of AD-T and BD-53 was used as a positive control, and the combination of empty AD and BD was used as a negative control. (**B**) Co-immunoprecipitation assay for the interaction between EqCmu and EqPdt. The total proteins (input) of *N. benthamiana* leaves expressing tested proteins were incubated with anti-GFP beads, and then the elution (IP) of anti-GFP beads was analyzed by Western blot using anti-GFP and anti-Flag antibodies. The GFP and GUS-Flag were used as negative controls. (**C**) BIFC assay for the interaction between EqCmu and EqPdt. EqCmu and EqPdt fused with C’-YFP and N’-YFP, respectively, were co-expressed in *N. benthamiana* leaves. YFP signals represent the interaction of two proteins. Bars, 50 µm. (**D**) Pull-down assay to test the interaction between EqCmu and EqPdt. Two tested proteins purified *in vitro* (input) were incubated with anti-His beads, and then the elution from beads (pull-down) was analyzed by Western blot with anti-GST antibody.

The results presented above prompted us to investigate whether EqCmu interacts with EqPdt to function. The yeast two-hybrid (Y2H) assay showed that yeast strains transformed with the combination of EqCmu-AD and EqPdt-BD grew on SD-LWHA plates and exhibited α-galactosidase activity (Fig. 7A). A co-immunoprecipitation assay was conducted using the co-expressed EqCmu-GFP and EqPdt-Flag in *N. benthamiana*, where EqPdt-Flag was immunoprecipitated by EqCmu-GFP (Fig. 7B). A bimolecular fluorescence complementation (BiFC) assay was performed, and the co-expression of EqCmu-C’YFP and EqPdt-N’YFP in *N. benthamiana* leaves yielded strong YFP signals in the cells (Fig. 7C). The results above suggest an interaction between EqCmu and EqPdt. Consistently, in a pull-down assay, EqPdt-GST was pulled down by EqCmu-His *in vitro*, and the addition of either infected or non-infected *H. brasiliensis* leaf cytosolic extract enhanced this interaction (Fig. 7D), implying that EqCmu may interact with EqPdt more intensively within host cells.

### EqCmu and EqPdt can be degraded in plant cells and mutually enhance the stability of each other

**Fig 8:**
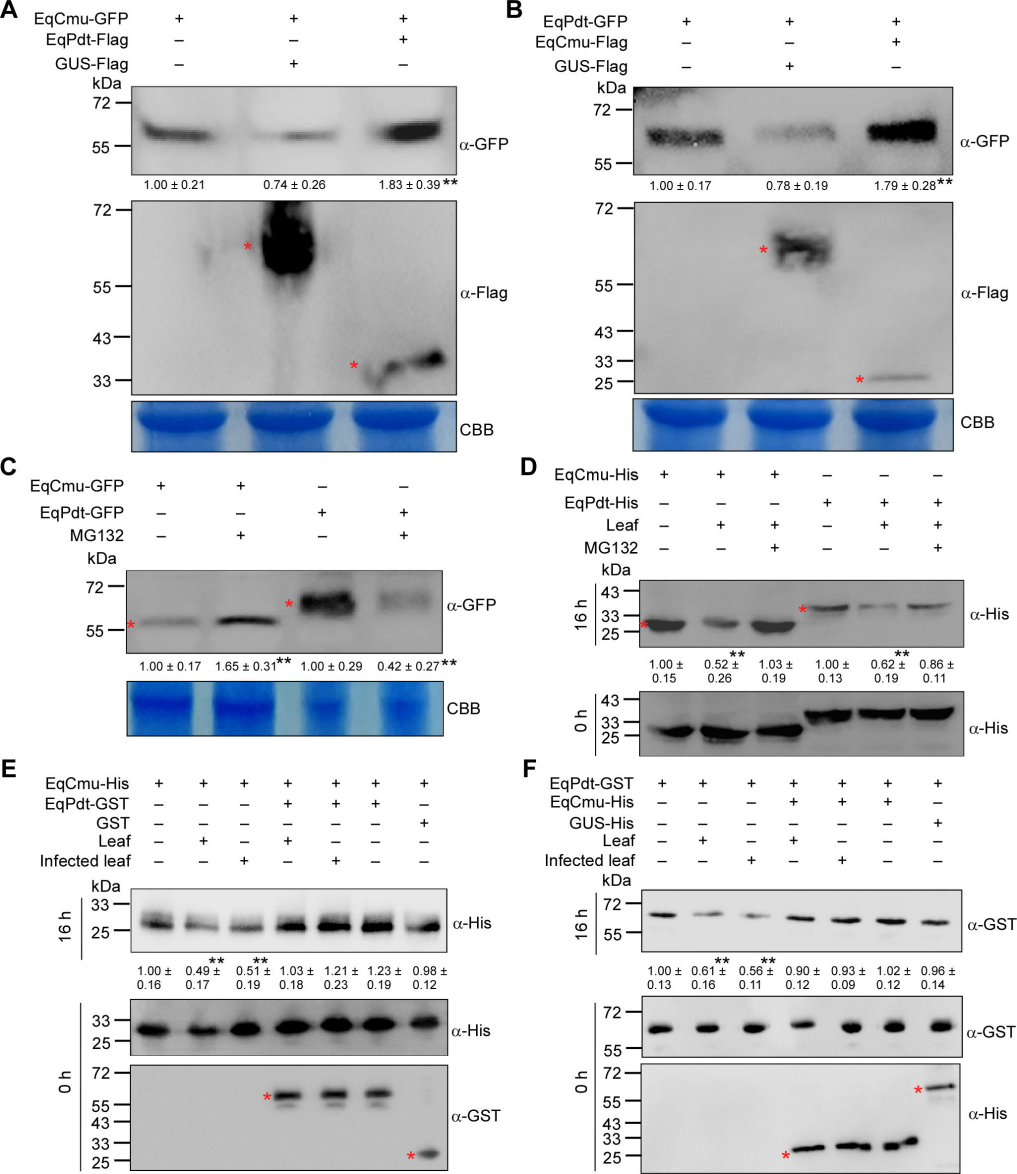
The destabilization of EqCmu or EqPdt by the plant was inhibited when these two proteins were present. (**A**) Analysis of the protein abundance of EqCmu-GFP expressed in *N. benthamiana* leaves. GUS-Flag was used as a control. Red asterisks indicate bands of GUS-Flag and EqPdt-Flag. In panels **A–C**, Coomassie brilliant blue (CBB) staining of total leaf extract was used as loading controls. In panels **A–F**, the values of band intensities were calculated from three independent experiments. Significant differences (*P* < 0.01) were analyzed using two-tailed Student’s *t*-test and indicated by black asterisks. (**B**) Analysis of the protein abundance of EqPdt-GFP expressed in *N. benthamiana* leaves. GUS-Flag was used as a control. Red asterisks indicate the bands of GUS-Flag and EqCmu-Flag. (**C**) Analysis of the protein abundance of EqCmu-GFP and EqPdt-GFP expressed in *N. benthamiana* leaves with or without 10 µM MG132 treatment. Red asterisks indicate the bands of EqCmu- and EqPdt-GFP. (**D**) Analysis of the protein abundance of EqCmu- and EqPdt-His at 0 h and after 16 h incubation with *H. brasiliensis* leaf cytosolic extract and MG132. Red asterisks indicate the bands of EqCmu- and EqPdt-His. (**E**) Analysis of the protein abundance of EqCmu-His at 0 h and after 16 h incubation with infected or non-infected *H. brasiliensis* leaf cytosolic extract and with EqPdt-GST or GST. Red asterisks indicate the bands of EqPdt-GST and GST. (**F**) Analysis of the protein abundance of EqPdt-GST at 0 h and after 16 h incubation with infected or non-infected *H. brasiliensis* leaf cytosolic extract and with EqCmu-His or GUS-His. Red asterisks indicate the bands of EqCmu-His and GUS-His.

Using *N. benthamiana* to express recombinant EqCmu and EqPdt, we found that the EqCmu-GFP signal was more intense in leaves co-expressing EqCmu-GFP and EqPdt-Flag compared with those co-expressing EqCmu-GFP and GUS-Flag (Fig. 8A). Similarly, the EqPdt-GFP signal was more intense in leaves co-expressing EqPdt-GFP and EqCmu-Flag than in those co-expressing EqPdt-GFP and GUS-Flag (Fig. 8B). Based on these results, we assume that EqCmu and EqPdt exhibit increased stability when they interact with each other.

We investigated whether the stabilities of EqCmu and EqPdt were affected by degradation through the UPS. Western blot analysis revealed that in *N. benthamiana* leaves expressing EqCmu-GFP alone, the signal intensity of EqCmu-GFP increased in the presence of MG132, an inhibitor of the 26S proteasome. In contrast, the signal intensity of EqPdt-GFP decreased in the presence of MG132 (Fig. 8C). To simulate conditions in *H. brasiliensi*s leaf cells containing both EqCmu and EqPdt, we mixed purified EqCmu- or EqPdt-His protein with the cytosolic extract of *H. brasiliensis* leaf cells, followed by a 16 h incubation at 22℃. After incubation, the signal intensities of both EqCmu- and EqPdt-His decreased; however, this decrease was inhibited by the addition of MG132 (Fig. 8D). Based on these results, it is possible that EqCmu was degraded in a 26S proteasome-dependent manner in *N. benthamiana* or within the cytosolic extract of *H. brasiliensis*. Although EqPdt alone may be degraded by unknown pathways in *N. benthamiana*, it is likely that EqPdt was degraded by the 26S proteasome within the cytosolic extract of *H. brasiliensis*.

To determine whether the destabilization of EqCmu and EqPdt by the plant UPS is specific, we expressed various GFP-fused CMUs and PDTs, including UmCmu1, *H. brasiliensis* CMUs (HbCmu1 and HbCmu2), the only *N. benthamiana* CMU (NbCmu) or PDT (NbPdt) in *N. benthamiana*. Subsequently, we assessed the protein content with or without MG132 treatment. Except HbCmu1, the GFP-fused CMUs and NbPdt exhibited stability, and their levels were not affected by MG132 treatment (Fig. S10). This suggests that the plant UPS cannot recognize all members of CMUs or PDTs.

Furthermore, we attempted to assess the stabilization of EqCmu and EqPdt by each other using a plant-fungal protein incubation assay. EqCmu-His and EqPdt-GST were incubated with the leaf cytosolic extract of *H. brasiliensis*. After the incubation, only EqCmu-His or EqPdt-GST was degraded in the leaf cytosolic extract (Fig. 8E, lanes 1–3; Fig. 8F, lanes 1–3). The degradation of EqCmu-His was inhibited by the addition of EqPdt-GST (Fig. 8E, lanes 4–6). Similarly, the degradation of EqPdt-GST was inhibited by the addition of EqCmu-His (Fig. 8F, lanes 4–6).

### The ubiquitination of EqCmu and EqPdt was suppressed by each other

**Fig 9:**
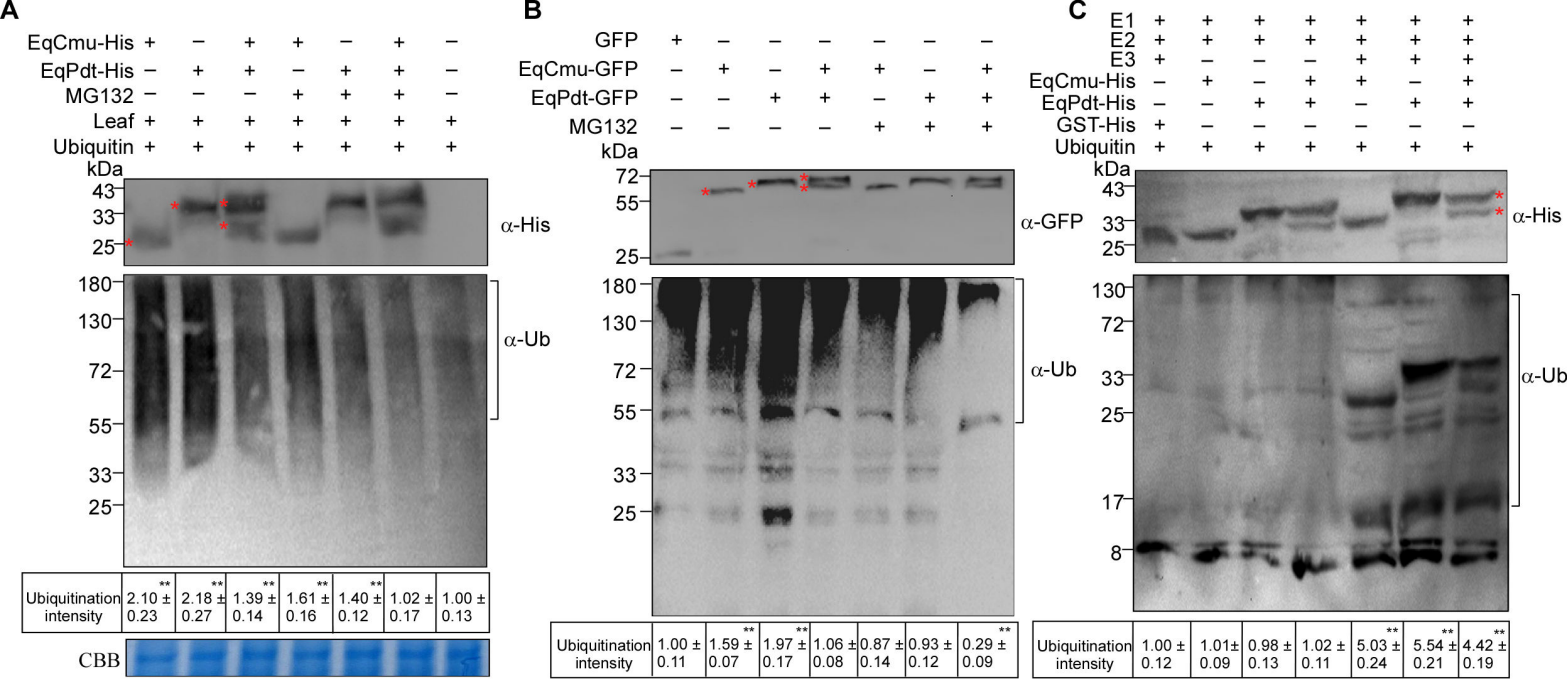
The interaction between EqCmu and EqPdt inhibited the ubiquitination of these two proteins. (**A**) Analyzing the ubiquitination levels of EqCmu-, EqPdt-His, or both after 4 h incubation with *H. brasiliensis* leaf cytosolic extract, MG132, and ubiquitin. Red asterisks indicate the bands of EqCmu- and EqPdt-His. Ubiquitinated proteins were indicated and analyzed with anti-ubiquitin (ɑ-Ub) antibody. In panels A–C, the values of ubiquitination intensities in the tables were calculated from three independent experiments. Significant differences (*P* < 0.01) are analyzed using two-tailed Student’s *t*-test and indicated by black asterisks. (**B**) Analyzing the ubiquitination levels of EqCmu-, EqPdt-GFP, or both expressed in *N. benthamiana*. Asterisks indicate the bands of EqCmu- and EqPdt-GFP. Ubiquitinated proteins were indicated and analyzed with anti-ubiquitin (ɑ-Ub) antibody. (**C**) Analyzing the ubiquitination levels of EqCmu-, EqPdt-His, or both after 4 h incubation with recombinant human UBE1 (E1), UBE2D1 (E2), UBE3 (E3), and ubiquitin protein. Asterisks indicate the bands of EqCmu- and EqPdt-His. Ubiquitinated proteins were indicated and analyzed with anti-ubiquitin (ɑ-Ub) antibody.

Because we found that EqCmu and EqPdt can stabilize each other to prevent degradation through the 26S proteasome, we incubated EqCmu-, EqPdt-His, or both with *H. brasiliensis* leaf cytosolic extract for 4 hours and determined the ubiquitination levels in the incubation mixtures. EqCmu- or EqPdt-His could be ubiquitinated after the incubation; however, the ubiquitination levels of EqCmu- and EqPdt-His were lower when these two proteins were combined (Fig. 9A, lanes 1–3). Interestingly, the addition of MG132 decreased the ubiquitination levels in each reaction compared with those without MG132 (Fig. 9A, lanes 4–6). This suggests that the inhibition of the 26S proteasome affected the activity of E3 ubiquitin ligases. Similarly, when EqCmu-, EqPdt-GFP, or both were expressed in *N. benthamiana* leaves, the ubiquitination levels of EqCmu- and EqPdt-GFP were lower in leaves co-expressing both proteins compared with those in leaves expressing EqCmu- or EqPdt-GFP alone (Fig. 9B). Notably, although MG132 induced EqPdt-GFP degradation in *N. benthamiana* (Fig. 8C), EqPdt-GFP remained ubiquitinated (Fig. 9B).

To further demonstrate that the interaction between EqCmu and EqPdt affects the ubiquitination of either EqCmu or EqPdt, we conducted an *in vitro* ubiquitination assay. In this assay, EqCmu- and EqPdt-His were incubated with recombinant human E1, E2, and E3 proteins. The ubiquitination levels in the incubation mixture containing both EqCmu- and EqPdt-His were lower than those observed with EqCmu- or EqPdt-His alone (Fig. 9C). Taken together, EqCmu and EqPdt can mutually protect each other from ubiquitination in plants.

## DISCUSSION

The data obtained from the NCBI database and previous studies show that CMUs and PDTs are widely distributed in plant pathogenic and non-pathogenic microorganisms. Although the enzymatic activities of CMUs or PDTs may allow them to catalyze aromatic amino acid biosynthesis in the shikimate pathway, this pathway is not present in animals, including nematodes. Pathogenic fungi, nematodes, and bacteria often encode secreted CMUs that can disturb plant metabolism and immunity (24, 25, 55–60). Pathogenic bacteria such as *Acidovorax citrulli* also produce CMU-PDT bifunctional proteins, which are non-secreted and promote the biogenesis of virulence factors, cell wall, and membranes during growth or plant infection (24). PDTs of oomycetes are rarely studied; however, a non-secreted PDT of the pathogenic oomycete *Phytophthora sojae* has been demonstrated to regulate nitrogen metabolism and pathogenic development (61). Non-pathogenic microbes, such as *E. coli*, *S. cerevisiae*, and *Aspergillus nidulans*, prefer to encode non-secreted CMUs and PDTs (53, 62–64). Previous studies on *E. coli* and *S. cerevisiae* only report that these enzymes are involved in the shikimate pathway (53, 63). Importantly, we note that the CMUs and PDTs of several powdery mildew fungi, besides *E. quercicola*, have been predicted to be unconventionally secreted proteins (Table S2). It is likely that powdery mildew fungi need secreted CMUs and PDTs to adapt their lifestyle.

**Fig 10:**
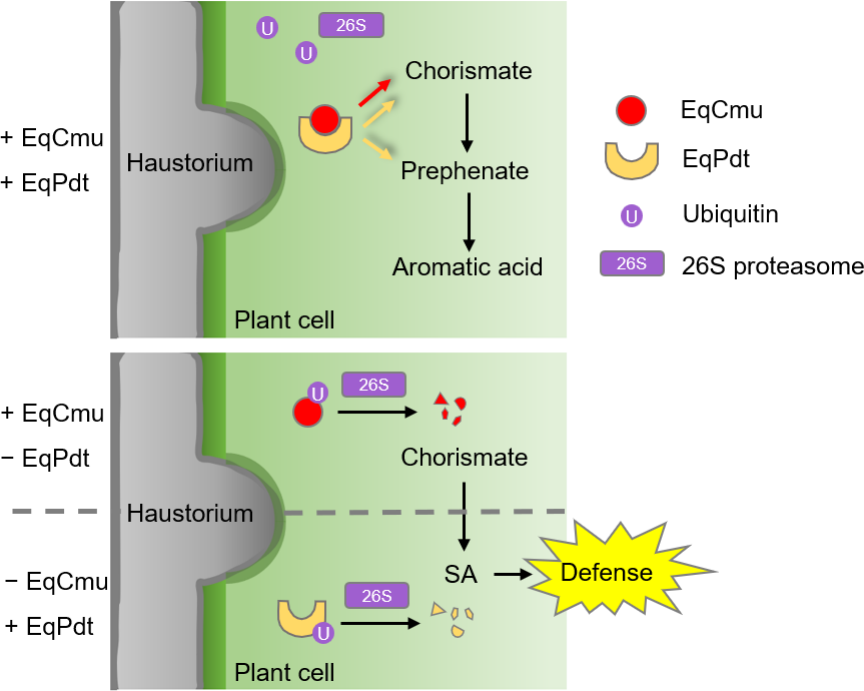
The model depicting how EqCmu and EqPdt function together to affect SA biosynthesis during infection. EqCmu and EqPdt can convert chorismate to aromatic acid but not SA. The interaction between EqCmu and EqPdt prevents the recognition of these fungal effectors by the plant UPS. When EqCmu or EqPdt functions alone, EqCmu or EqPdt is ubiquitinated and degraded by the 26S proteasome, leading to the activation of SA-mediated immunity.

Our study suggests that the powdery mildew effector proteins EqCmu and EqPdt function together to directly disrupt SA signaling (Fig. 10). Translocation into host cells is a prerequisite for EqCmu and EqPdt function. Although these two proteins have no predicted signal peptides, unlike *U. maydis* Cmu1 (UmCmu1), bioinformatics analysis predicted them as unconventionally secreted proteins (Table S1). The secretion of these two proteins was verified by performing the yeast invertase secretion and *M. oryzae* protein secretion assays. When EqCmu and EqPdt were expressed in the yeast, they could induce invertase secretion (Fig. 5A). When they were expressed in *M. oryzae*, they were delivered into BICs during barley infection (Fig. 5C). We also investigated effector translocation following secretion. We exogenously supplied recombinant purified EqCmu- and EqPdt-His to a strain in *H. brasiliensis* leaves and detected the subcellular localization of these proteins. Using immunofluorescence staining, we observed that EqCmu- and EqPdt-His accumulated in the EHM (Fig. S4); therefore, the exogenously supplied proteins might have entered the pathways for effector translocation in the plant. We detected EqCmu- and EqPdt-His, but not the control GST-His, in the PM and cytosolic fractions of the protoplasts isolated from those leaves via Western blotting, revealing that these two effector proteins can adhere to the plant PM, followed by their entry into the host cells during infection (Fig. 5E). Additionally, the accumulation of EqCmu and EqPdt in BICs during rice infection by *M. oryzae* also implies the entry of these two proteins into the plant cytosol. Many cytosolic effectors of *M. oryzae* were found to accumulate in BICs prior to plant cell entry. Recently, clathrin-mediated endocytosis in plants mediated the uptake of effectors of *M. oryzae* and *Pythophthora infestans*, an oomycete pathogen (49, 65). Plant endocytic vesicles for effector transport accumulate in BICs or the EHM during infection by these pathogens (49, 65). We hypothesize that endocytosis pathways probably transport EqCmu and EqPdt into plant cells following the establishment of the EHM.

It was previously found that EqCmu complemented the growth defect phenotype of a yeast CMU-deficient mutant (Δ*ScAro7*) (31). Here, EqPdt complemented the growth defect phenotype of a PDT-deficient mutant (Δ*ScPha2*). In the *in vitro* enzymatic activity assays, both EqCmu and EqPdt converted chorismate to aromatic amino acids, and this conversion was dependent on key catalytic residues in these enzymes (Fig. 6; Fig. S9). Additionally, the phenylalanine content was elevated in *H. brasiliensis* leaves inoculated with the *EqCmu*- and *EqPdt*-silenced strains. The results demonstrate the enzymatic activity of EqCmu and EqPdt.

These enzymatic activities enable EqCmu and EqPdt to inhibit host SA biosynthesis. The expression of EqCmu, EqPdt, or both in *N. benthamiana* reduced the SA level (Fig. 4D). *EqCmu*- and *EqPdt*-silenced strains, as well as a strain with both genes silenced, induced SA accumulation and SA signaling in host leaves (Fig. 4A and B). Moreover, the DPI treatment assay indicated that EqCmu and EqPdt are important for suppressing host immunity. Administering the NADPH oxidase inhibitor DPI as a treatment to the plant largely restored the infection ability of *EqCmu*- and *EqPdt*-silenced strains as well as the strain with both genes silenced (Fig. 3E; Fig. S2). NADPH oxidase deactivation by DPI is supposed to deactivate plant immunity. Recently, NADPH oxidases, such as RbohD and RbohF, were demonstrated to be key components in the signaling pathways of pathogen-associated molecular pattern- and effector-triggered immunity (44, 66).

Our results reveal that EqCmu and EqPdt are substrates of the plant UPS. Inhibition of the 26S proteasome through MG132 treatment increased the EqCmu content in transformed *N. benthamiana* leaves (Fig. 8C). Additionally, MG132 treatment inhibited the degradation of EqCmu or EqPdt after incubation with a cytosolic extract from *H. brasiliensis* leaves (Fig. 8D). Furthermore, the ubiquitination of EqCmu and EqPdt was observed after incubation with the *H. brasiliensis* leaf cytosolic extract or in *N. benthamiana* leaves (Fig. 9A and B). However, when we expressed other CMUs or PDTs in *N. benthamiana*, we found that the UPS did not affect the CMUs from *U. maydis*, *H. brasiliensis*, and *N. benthamiana*, as well as NbPdt (Fig. S10). A possible explanation for this observation is that certain CMUs or PDTs may have evolved mutations that confer resistance to UPS-mediated degradation. This adaptation likely serves as a strategy to enhance protein stability, which is advantageous for the physiological processes within an organism. Additionally, while we found that EqCmu and EqPdt can be targeted by the UPS, we identified a potential mechanism by which these two proteins maintain stability; they form a heterodimer to evade ubiquitination.

We first identified the interaction between these two proteins using Y2H, co-immunoprecipitation, BiFC, and His-pull-down assays. In a pull-down assay, the addition of the *H. brasiliensis* cytosolic extract enhanced the interaction, possibly due to the presence of plant proteins or chemical molecules that promote this interaction (Fig. 7). Therefore, EqCmu and EqPdt may interact more intensely after translocation into host cells. Additionally, in our assays, EqCmu and EqPdt were more stable when co-expressed in *N. benthamiana* or when added together after incubation with the *H. brasiliensis* cytosolic extract (Fig. 8). The ubiquitination levels of EqCmu and EqPdt decreased when these two proteins were present together (Fig. 9). We propose that the interaction between EqCmu and EqPdt enhances their stabilization in plant cells, although these results were obtained from *in vitro* assays only. In most cases, the UPS serves as an important regulatory component of plant immunity. Studies on the rice blast fungus effector AvrPiz-t have revealed that the plant UPS can directly counteract alien effectors (67, 68). Two rice E3s, APIP6 and APIP10, were found to ubiquitinate AvrPiz-t and promote AvrPiz-t degradation via the 26S proteasome; however, AvrPiz-t could degrade APIP6 and APIP10, in return, to disturb the UPS (67, 68). Here, we provide additional information on how fungal effectors escape recognition by the plant UPS (Fig. 10). We also hypothesize that the interaction between EqCmu and EqPdt may cover the ubiquitination sites of these two proteins.

## MATERIALS AND METHODS

### Growth conditions of experimental plants and the powdery mildew fungus

*N. benthamiana* and the *H. brasiliensis* cultivar Reyan 7-33-97 were planted in an experimental greenhouse with a 16/8 hours light/dark cycle at 24℃. Seven-day-old Reyan 7-33-97 specimens were infected by the powdery mildew *E. quercicola* (HO-73 strain) for multiplication.

### dsRNA application

dsRNA was synthesized using a T7 RNAi transcription kit (Vazyme). Fluorescein-labeled dsRNA was synthesized using dUTP-fluorescein (Thermo). Seven-day-old *H. brasiliensis* leaves were inoculated with fresh powdery mildew conidial suspension (1 × 10^5^ spores/mL), followed by dsRNA treatment. The dsRNA solutions (20 ng/uL) were dropped onto the inoculation sites to immerse the conidia, and the inoculated plants were kept in the greenhouse until the leaf surfaces were dried. dsRNAs were applied to the inoculated sites again at 48 hpi. The total RNA of a strain treated with dsRNA was collected, and the targeted gene transcripts were analyzed by performing qRT-PCR.

### Assays of lesion area measure and conidiation

For the SANa and BTH treatment assays, 7-day-old *H. brasiliensis* leaves were treated with SANa or BTH solution. After 24 hours, each leaf was sprayed with 200 µL conidial suspension (1 × 10^5^ spores/mL) for inoculation. The lesion area per leaf was calculated using ImageJ software (1.49 V) to analyze the photographs. For the pathogenicity assays with strains treated with dsRNA, the lesion area of inoculation sites was also calculated using this software. In the conidiation assay, leaf discs (1 cm^2^ per leaf disc) obtained from the infected leaves were used for conidium collection. The conidia on the surface of each disc were washed off using distilled water and resuspended in 100 µL distilled water. The conidia were quantified with a hemocytometer under a dissecting microscope.

### qRT-PCR analysis

The total RNAs from *E. quercicola* or *H. brasiliensis* were extracted using TRIzol reagent (6) and reverse transcribed into complementary DNA (cDNA) using a reverse transcription kit (Vazyme). qRT-PCR analysis was conducted using the SYBR Green Realtime PCR Master Mix (Vazyme) on the QuantStudio 12K Flex system (Thermo) under the following PCR conditions: 95°C for 30 seconds and 40 cycles at 95°C for 5 seconds and 60°C for 30 seconds. Primers are listed in Table S4. The *EqEF-1a* and *HbActin* genes were used as reference genes of *E. quercicola* and *H. brasiliensis*, respectively. Three independent experiments were conducted, producing similar results.

### Determination of SA concentration

To measure the SA levels and conjugates in *H. brasiliensis* and *N. benthamiana* leaves, 90 mg of leaf tissue per sample was collected and ground in liquid nitrogen. The organic phase containing SA and SA conjugates was dried and then suspended in 1  mL of methanol. Ten microliters of the filtered suspension was loaded onto a liquid chromatography–mass spectrophotometry platform (Agilent Technologies Inc) for SA level measurement, according to the method developed by Liu et al. (12). The suspension was separated on a C18 analytical column (150 × 2.1 mm; 5 µm) at a flow rate of 0.3  mL/min. The SA concentration was quantified according to the measured standard curve (*y* = 6,674.9*x* – 487.1; *R*2 = 0.9999, retention time = 10.23 minutes).

### Measurement of phenylalanine content in *H. brasiliensis* leaves

Leaf tissue (10 mg) was collected per sample and ground in liquid nitrogen. After hydrolysis and derivatization, phenylalanine concentration was measured by conducting chromatographic analysis in a high-performance liquid chromatography (HPLC)-integrated system (WUFENG), according to the method developed by Pimentel et al. (69). The suspension was separated on a DAICEL CHIRALPAK AD-H chiral column (250 mm × 4.6 mm, 5 µm) with 1.0  mL/min flow rate at 35°C. The phenylalanine concentration was quantified according to the measured standard curve (*y* = 1.0123*x* – 4.5503; *R*2 = 1.000, retention time = 30.173 minutes).

### Protein expression, extraction, and purification

*Agrobacterium*-mediated transformation was conducted to express the GFP- and Flag-tagged proteins used for the assays with *N. benthamiana*. The encoding sequences were cloned into pBin4-GFP and pCambia-Flag vectors. All primers used in sequence amplification for vector construction are listed in Table S4. *Agrobacterium* GV3101 strains carrying the recombinant plasmids were washed and resuspended in 10 mM MgCl_2_. The bacterial suspension (OD_600_ = 0.4) was infiltrated into the leaves of 4-week-old *N. benthamiana*. Total leaf proteins were extracted using a lysis buffer (150 mM NaCl, 50 mM Tris-HCl, pH 7.5, 1.0% (vol/vol) NP-40, 1 mM PMSF, and 0.1% (vol/vol) protease inhibitor cocktail). *H. brasiliensis* leaf cytosolic extract was obtained using the Plant Cytosolic Protein Isolation Kit (Invent). To obtain purified His- and GST-tagged proteins *in vitro, E. coli* BL21 (DE3) strains carrying recombinant plasmids were incubated at 37°C in lysogeny broth medium to OD_600_ = 0.6 to 0.8; afterward, they were incubated with 0.8 mM IPTG for 16 hours at 16°C. Bacterial cells were lysed in a lysis buffer at pH 7.5 (20 mM NaH_2_PO_4_, 10 mM imidazole, and 300 mM NaCl) through ultrasonication; subsequently, the supernatant was obtained and incubated with Ni-NTA or GST resin beads (Thermo) at 4°C for 4 hours. The beads were washed six times with a wash buffer (50 mM NaH_2_PO_4_, 20 mM imidazole, 300 mM NaCl, 1 mM PMSF, pH 7.5). The His-tagged proteins from Ni-NTA were eluted with an elution buffer-1 (50 mM NaH_2_PO_4_, 250 mM imidazole, 300 mM NaCl, pH 7.5), and GST-tagged proteins from GST resin beads were eluted with an elution buffer-2 (50 mM Tris-HCl, 10 mM glutathione, reduced [GSH], pH 8.0).

### Isolation of the plasma membrane and cytosolic fractions from *H. brasiliensis* leaf protoplasts

*H. brasiliensis* mesophyll protoplast isolation was carried out as previously described (40). Briefly, the young leaves were soaked in 1.5% cellulase R10 and 0.3 % macerozyme R10 for digestion reaction for 5 hours, and protoplasts were released and suspended in W5 solution (154 mM NaCl, 125 mM CaCl_2_, 5 mM KCl, 5 mM glucose, 2 mM MES, pH 5.7). The plasma membrane and cytosolic proteins were isolated using the Plant Plasma Membrane Protein Isolation Kit (Invent) (44). In this method, protoplasts were first sensitized, homogenized, and passed through a specialized filter cartridge that allows homogenates to pass through with a zigzag path. The PM was ruptured into a range of predefined size, and the cytosolic fraction was separated during the process.

### Co-immunoprecipitation assay using *N. benthamiana*

The EqCmu- and EqPdt-encoding sequences were cloned into pBin4-GFP and pCambia-Flag vectors to construct pBin4-*EqCmu*-GFP and pCambia-*EqPdt*-Flag plasmids. Two Agrobacterium GV3101 strains carrying these recombinant plasmids were co-infiltrated into *N. benthamiana* leaves to express EqCmu-GFP and EqPdt-Flag. Subsequently, the total leaf proteins were extracted and incubated with GFP beads (Chromotek) at 4°C for 4 hours. After incubation, the beads were washed six times with a wash buffer (50 mM HEPES at pH 7.5, 150 mM NaCl, 10% glycerol, 5 mM EDTA, 1 mM DTT, and 0.1% Triton X-100) and then boiled in a sodium dodecyl sulfate (SDS) loading buffer for 5 minutes. The proteins (10 µg per sample) were separated by using 12% SDS-PAGE gel and detected through Western blotting using anti-GFP (1:5,000; Abcam) and anti-DDDK monoclonal antibodies (1:5,000; Abcam).

### Yeast gene-knockout and transformant screening

A single-step gene deletion method was used to knockout the *ScPha2* gene from the yeast strain YPG30, as previously described (31). YPG30 was transformed with the PCR product containing the *His3* gene and the 60 bp flanking regions responsible for homologous recombination in *S. cerevisiae*. After transformation, colonies were selected on a medium lacking histidine. To transform Δ*ScPha2* with *ScPha-HA* and *EqPdt-HA*, the recombinant pYES2 constructs carrying these two sequences were introduced into Δ*ScPha2*. Subsequently, the transformants were verified based on their growth on a Phe-lacking synthetic defined medium and through Western blot to detect the expressions of ScPha-HA and EqPdt-HA.

### Y2H assay

The EqCmu- and EqPdt-encoding sequences were cloned into pGADT7 or pGBKT7 vectors, and the recombinant plasmids were introduced into the yeast strain Y2HGold. The strains were cultured on an SD medium lacking leucine and tryptophan (SD-LW) at 30℃ for 3 days for transformant selection, and then the transformants were transferred to an SD medium lacking leucine, tryptophan, histidine, and adenine (SD-LWHA) with X-α-gal addition for assaying the interaction between EqCmu and EqPdt.

### Bimolecular fluorescence complementation (BiFC) assays

pSPYCE-*EqCmu* and pSPYNE-*EqPdt* plasmids were constructed, and the resultant plasmids were allowed to express EqCmu-C’YFP and EqPdt-N’YFP in *N. benthamiana* leaves through *Agrobacterium*-mediated transformation. YFP signals were detected using a fluorescence microscope 48 hours after *Agrobacterium* infiltration (excitation: 514 nm; emission: 525–575 nm).

### *In vitro* pull-down assays

The pET28a-*EqCmu*-His and pGEX6p-*EqPdt*-GST constructs were introduced into *E. coli* BL21 cells for EqCmu-His and EqPdt-GST production, and these proteins were purified as described above. EqCmu-His (10 µg) was incubated with anti-His beads (Thermo) at 4℃ for 4 hours. Afterward, the beads were washed at least six times with the wash buffer and incubated with EqPdt-GST (5 µg) and leaf cytosolic extract (30 µg) at 4°C for 2 hours. The beads were collected and washed at least six times with the wash buffer, resuspended in 1× SDS loading buffer, and boiled at 100℃ for 5 minutes; subsequently, they were analyzed by immunoblotting with anti-His (Abcam, 1:5,000) and anti-GST (Abcam, 1:5,000) antibodies.

### Protein localization analysis in *M. oryzae* pathosystem

The EqCmu-, EqPdt-, or MoCmu-coding sequence was fused with the native promoter of MoCmu and was cloned into the pJNARG-GFP vector that carries geneticin-resistant gene. The BAS1-coding sequence was fused with its native promoter and mCherry and was cloned into the pXY203 vector that carries hygromycin-resistant gene (50, 70). A recombinant pJNARG-GFP and a recombinant pXY203 plasmid were co-transformed into *M. oryzae* strain Guy11 via PEG-mediated transformation (71). Transformants were screened using 350 µg/mL of geneticin and 400 µg/mL of hygromycin. The fresh conidia of transformants were allowed to infect 7-day-old barley leaves at 28℃. The infected epidermal cells of leaves were examined under a confocal laser scanning microscope (GFP/mCherry excitation: 488/580 nm; emission: 507/610 nm). Linescan analyses of the microscopic images were conducted with ImageJ software (1.49 V).

### Immunofluorescence assay

The *H. brasiliensis* leaf discs with applying EqCmu- and EqPdt-His were immobilized with methanol. The leaves were washed with 1× PBS and immersed in a blocking buffer (3% BSA in PBST), and then incubated in 1% BSA in PBST buffer containing anti-His antibodies at 4°C for 7 hours. After washing with PBST thrice, the samples were incubated with the Alexa Fluor 488-conjugated secondary antibody (1:500, Abcam) for 1 hour at 4°C. After washing with 1× PBS at 26°C for 15 minutes, fluorescence signals were detected using a confocal laser scanning microscope (Alexa Fluor 488 excitation: 490 nm; emission: 525 nm).

### Ubiquitination assays

The purified EqCmu- and EqPdt-His proteins were obtained as described previously. The purified EqCmu-His, EqPdt-His, or both (3 µg of each protein) were mixed with 50 ng of ubiquitin-activating enzyme (UBE1) (YEASEN), 200 ng of ubiquitin-conjugating enzyme (UBE2) (YEASEN), 1 µg of ubiquitin-ligase enzyme (UBE3) (YEASEN), and 1 µg of ubiquitin (Ub) (YEASEN), and were incubated in a reaction buffer (50 mM Tris-HCl, pH 7.4, 10 mM MgCl_2_, 5 mM ATP, 2 mM DTT) to a final volume of 30 µL at 37°C in the thermomixer for 4 hours (72). The reaction was terminated by adding an SDS-loading buffer and boiling for 5 minutes. The proteins were analyzed through immunoblotting using an anti-ubiquitin antibody (1:1,000, Proteintech).

To detect the ubiquitination of EqCmu and EqPdt in the reaction with *H. brasiliensis* cytosol extract, the purified EqCmu-His, EqPdt-His, or both (3 µg of each protein) were mixed with leaf cytoplasmic protein (30 µg) and 1 µg of ubiquitin (Ub) (YEASEN), and incubated in a reaction buffer (50 mM Tris-HCl, pH 7.4, 10 mM MgCl2, 5 mM ATP, 2 mM DTT) to a final volume of 200 µL at 37°C in the thermomixer for 4 hours. The reaction was stopped by adding the SDS-loading buffer and boiling at 100°C for 5 minutes. The proteins were analyzed through immunoblotting using an anti-ubiquitin antibody (1:1,000, Proteintech).

## Data Availability

The sequence data for cDNAs from this study can be found in the GenBank database under the following accession numbers: *EqCmu* (MK391402.1), *EqPdt* (MK391403.1), *EqIsc1* (MN563786.1), *EqTub2* (KT923674.1), *S. cerevisiae Pha2* (NP_014083.2), *Streptococcus mutans Pdt* (WP_002261940.1), *Chlorobaculum tepidum Pdt* (WP_010933330.1), *Escherichia coli CM/PDT* (WP_000200140.1), MoCmu (XP_003714705.1), *HbPR1* (XM_021821935.1), *HbActin* (GU270586.1), *HbNPR1* (KF753695.1), and *HbICS2* (XM_021821468.1).

## SUPPLEMENTAL MATERIAL

Supplemental Figures mbio.03959-24-s0001.docx Figure S1 to S10.

Supplemental Tables mbio.03959-24-s0002.docx Tables S1 to S4.

ASM does not own the copyrights to Supplemental Material that may be linked to, or accessed through, an article. The authors have granted ASM a non-exclusive, world-wide license to publish the Supplemental Material files. Please contact the corresponding author directly for reuse.
