# Supplementary material for: Plant salicylic acid signaling is inhibited by a cooperative strategy of two powdery mildew effectors
Source: mBio. 2025 Mar 17;16(4):e03959-24. doi: 10.1128/mbio.03959-24 (PMC11980547; doi:10.1128/mbio.03959-24)
Supplement: Supplemental Figures — Figure S1 to S10. [file mbio.03959-24-s0001.docx]

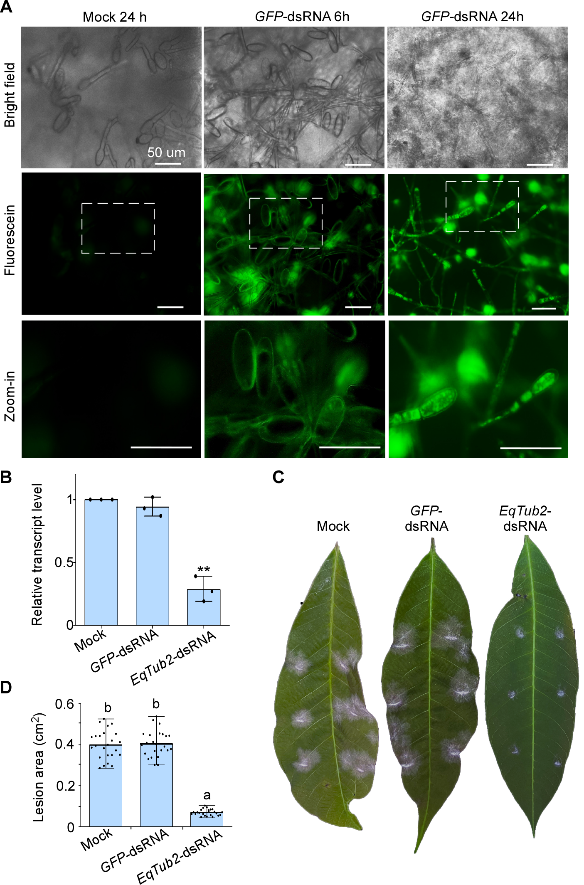


**Fig. S1 Exogenous dsRNA could be absorbed by and induce gene silencing in *E. quercicola*.**

**(A)** Exogenous *GFP*-dsRNA can be absorbed by *E. quercicola*. *GFP*-dsRNA labeled with fluorescein was generated and sprayed to *E. quercicola* colonies. Fluorescein signals were detected on the surfaces of conidia and hypha at 6 h post-treatment and inside the conidia and hypha at 24 h. Areas labeled by white squares were magnified in zoom-in panels. Mock, H_2_O treatment. Bars, 50 μm. **(B)** Relative transcript levels of *EqTub2 gene* in *E. quercicola*. Total RNA of the fungus was collected at 5 dpi. Bar chart showing the mean and SD with 3 biological replicates (n = 3 for each replicate). The mock was compared with each of other samples using two-tailed Student’s t-test. Significant differences (*P* < 0.01) are indicated by asterisks (**). **(C)** *E. quercicola* treated with *EqTub2*-dsRNA reduced pathogenicity. *H. brasiliensis* leaves were inoculated with *E. quercicola* conidia, followed by *EqTub2*-dsRNA treatment to the fungus. The photographs were taken at 5 dpi. **(D)** The quantification of lesion areas on *H. brasiliensis* leaves was shown. Bar chart show the means and SD (n = 24 from 3 independent experiments). One-way ANOVA with Tukey’s test was used to analyze statistical difference indicated by different letters (*P* < 0.01).


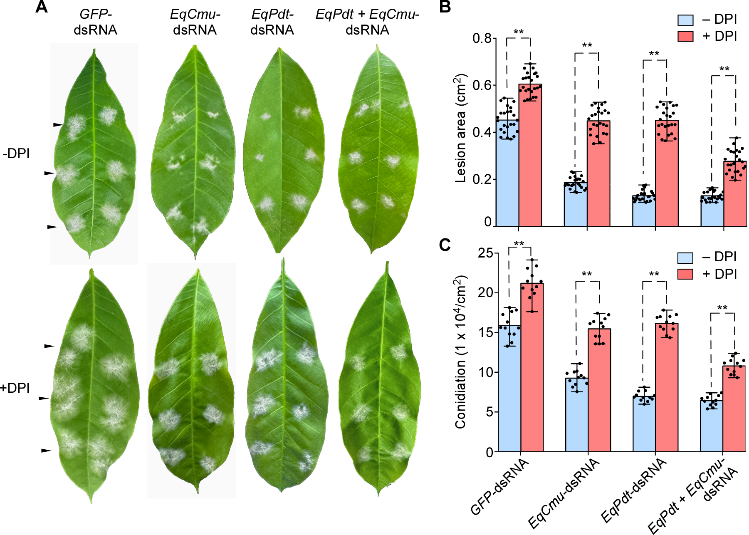


**Fig. S2 DPI application restored pathogenicity by the gene-silenced strains.**

**(A)** The photographs show pathogenicity by the gene-silenced strains in leaves with or without DPI application at 7 dpi. Arrows indicate inoculation sites. **(B)** Quantification of the lesion areas on *H. brasiliensis* leaves. Bar chart shows the means and SD (n = 24 from 3 independent experiments). **(C)** Quantification of conidia produced by the *E.* *quercicola* strains. Bar chart shows the means and SD (n = 12 from 3 independent experiments). In (B and C), The samples with DPI application were compared with these without DPI application using two-tailed Student’s t-test. Significant differences (P < 0.01) are indicated by asterisks (**).


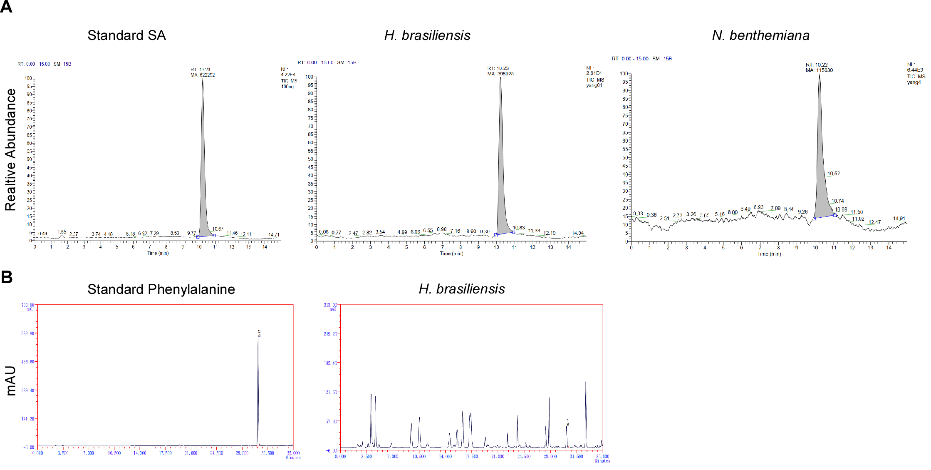


**Fig. S3 Chromatograms of LC-MS/MS and HPLC.**

**(A)** Representative LC-MS/MS chromatograms for SA measurements. These chromatograms show the LC-MS/MS analysis of standard SA and samples derived from *H. brasiliensis* leaves inoculated with *GFP*-dsRNA-treated *E. quercicola* and from *N. benthemiana* leaves expressing GFP. **(B)** Representative HPLC chromatograms of phenylalanine measurements. These chromatograms show the HPLC analysis of the standard phenylalanine and the sample derived from *H. brasiliensis* leaves inoculated with *GFP*-dsRNA-treated *E. quercicola*.


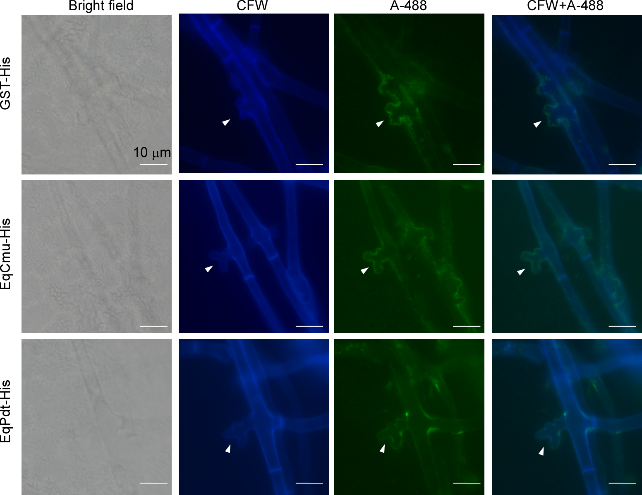


**Fig. S4** **Immunofluorescence assay to examine the protein applied to *E. quercicola* during infection.**

Immunofluorescence images show that exogenously applied EqCmu-His and EqPdt-His proteins (30 μg/ml) were translocated into leaf tissues during *E. quercicola* infection. The purified EqCmu- and EqPdt-His proteins were applied to leaf surfaces for 12 h, and the proteins were examined by immunofluorescence assay. The localizations of EqCmu- and EqPdt-His were detected by a primary anti-His antibody and visualized by Alexa Fluor 488-conjugated secondary antibody (A-488). Calcofluor White (CFW) was used as a fungal cell wall marker. White arrows indicate haustoria. Bars, 10 μm.


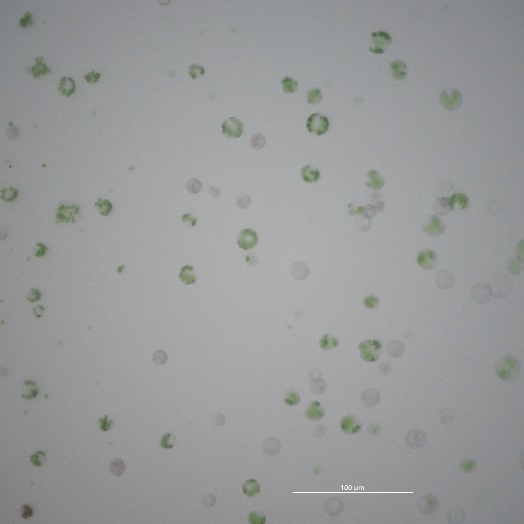


**Fig. S5** **The representative image of isolated *H. brasiliensis* mesophyll protoplasts.**

The protoplasts were isolated from *H. brasiliensis* mesophyll cells, and suspended in W5 solution for microscopic examination. Bar, 100 μm.


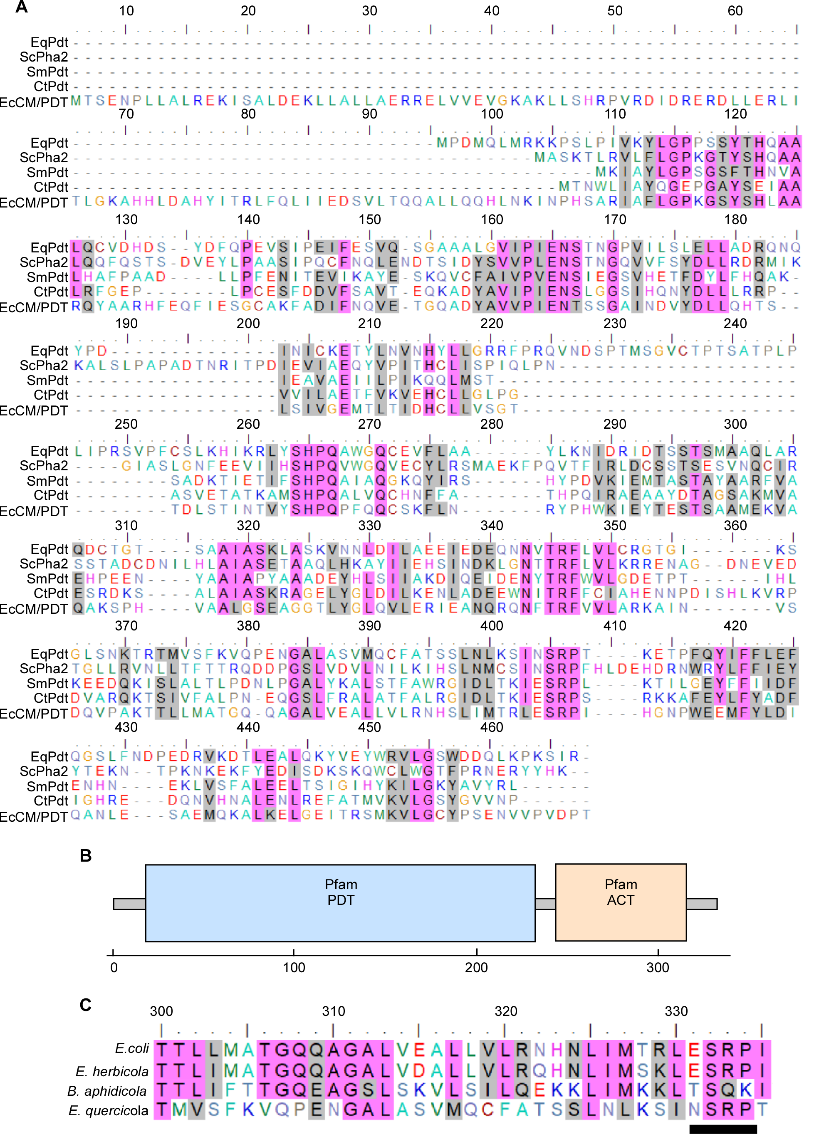


**Fig. S6** **Conserved residues and predicted protein domains in EqPdt**.

**(A)** Alignment of EqPdt (QDZ38302.1) with reported PDTs, including *S. cerevisiae* Pha2 (NP_014083.2), *Streptococcus mutans* Pdt (WP_002261940.1), *Chlorobaculum tepidum* Pdt (WP_010933330.1), and *Escherichia coli* CM/PDT (WP_000200140.1). Conserved residues are highlighted in pink (highly conserved) and gray (moderately conserved). **(B)** Protein domain analysis of EqPdt. EqPdt contains prephenate dehydratase (PDT) and ACT domains (ACT domains are linked to a wide range of metabolic enzymes regulated by amino acid concentration). **(C)** Alignment of the homologous region of the regulatory domains from *E. coli* PDT (P07022), *E. herbicola* PDT (Q02286), *Buchnera aphidicola* PDT (AJ239043) and EqPDT. The ESRP region were marked by black line.


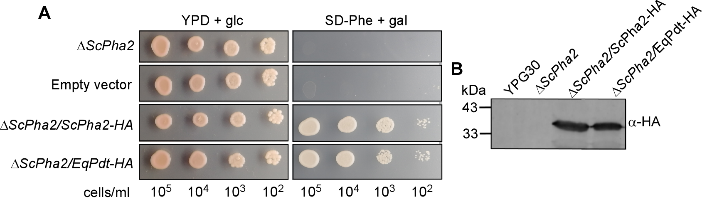


**Fig. S7** **The defect of *S. cerevisiae*** ***ScPha2* deletion mutant in phenylalanine synthesis could be rescued by EqPdt.**

**(A)** Growth of the strains on SD medium lacking phenylalanine was tested. The *EqPdt-HA*, and *ScPha2-HA* were expressed in the *S. cerevisiae ScPha2* deletion mutant strain Δ*ScPha2*, the growth of the strains on medium lacking phenylalanine was tested. *ScPha2–HA* and pYES2 empty vectors were used as positive and negative controls, respectively. YPD, nutrient-rich medium; glc, glucose; Gal, galactose; Phe, phenylalanine. **(B)** Western blotting of EqPdt-HA and ScPha2-HA in total proteins extracted from yeast strains using anti-HA antibody.


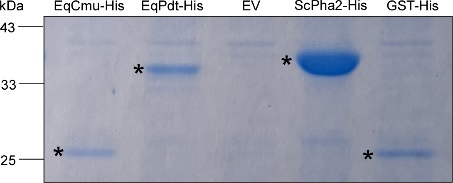


**Fig. S8** **Purification of EqCmu, EqPdt, ScPha2 and GST proteins.** Purified proteins derived from *E. coli* BL21 were analyzed by SDS-PAGE with Coomassie Brilliant staining. Asterisks indicate bands of EqCmu-, EqPdt-, ScPha2- and GST-His. EV: elution of Ni-NTA beads incubated with the cell lysate of a bacterial strain carrying the empty pET28a vector.


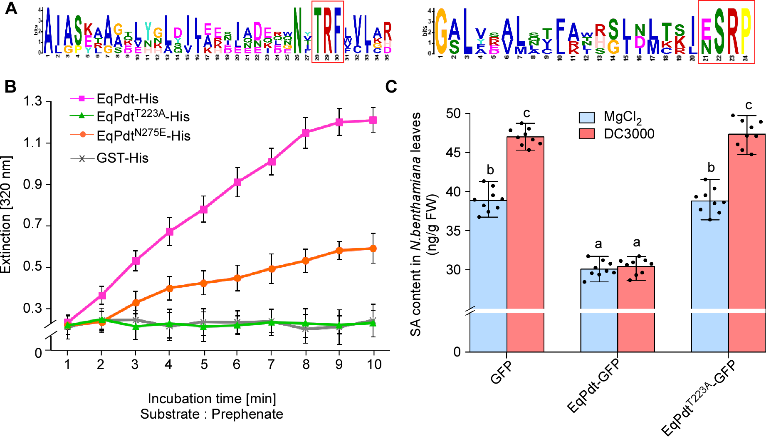


**Fig. S9 The** **identification of enzymatic activity sites of EqPdt.** **(A)** Multiple sequence alignment of PDTs from *S. cerevisiae*, *S. mutans*, *C. tepidum* and *E. coli*, using MEME tool (https://meme-suite.org/meme/tools/meme). Highly conserved TRF motif at the PDT domain and E/NSRP motif at the ACT domain are indicated by red boxes. **(B)** T223 and N275 are essential for enzymatic activity of EqPdt. The purified of EqPdt-, EqPdt^T223A^-, EqPdt^N275E^- and GST-His (5 μg of each protein) were incubated with prephenate, followed by the measurement of phenylpyruvate absorbance (320 nm) at the indicated time points (n = 9 from 3 independent experiments). **(C)** EqPdt^T223A^ did not inhibit salicylic acid biosynthesis in *N. benthamiana*. Leaf discs were collected for LC/MS-MS analysis at 48 h after DC3000-inoculation or infiltration with MgCl_2_ solution. SA content in *N. benthamiana* leaf tissues was measured using LC/MS-MS. Bar chart show the means and SD (n = 9 leaf discs from 3 independent experiments). One-way ANOVA with Tukey’s test was used to analyze statistical difference indicated by different letters (*P* < 0.01).


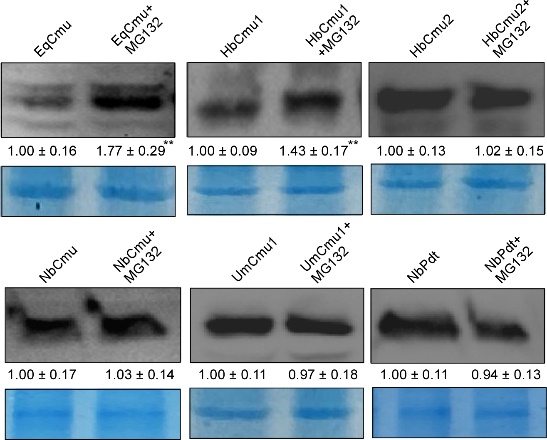


**Fig. S10** **The determination of protein abundance in *N. benthamiana* with or without MG132 treatment.**

Western blotting of EqCmu-GFP, HbCmu1-GFP, HbCmu2-GFP, NbCmu-GFP, UmCmu1-GFP and NbPdt-GFP expressed in *N. benthamiana* leaves with or without 10 μM MG132 treatment. Band intensities were calculated from three independent experiments. Significant differences (P < 0.01) are analyzed using two-tailed Student’s t-test and indicated by asterisks (**).
