# Supplementary material for: Plant salicylic acid signaling is inhibited by a cooperative strategy of two powdery mildew effectors
Source: mBio. 2025 Mar 17;16(4):e03959-24. doi: 10.1128/mbio.03959-24 (PMC11980547; doi:10.1128/mbio.03959-24)
Supplement: Supplemental Tables — Tables S1 to S4. [file mbio.03959-24-s0002.docx]

**Table. S1** EqPdt, EqIsc1 and MoCmu were predicted as non-classically secreted proteins.

| Protein | NN Score (SecretomeP 1.0f) | N-terminal signal peptide (SignalP 6.0) |
| --- | --- | --- |
| EqPdt | 0.632 | No |
| EqIsc1 | 0.829 | No |
| MoCmu | 0.842 | No |

**Note.** Secretory characteristics of these proteins were analyzed using SecretomeP 1.0f (http://www.cbs.dtu.dk/services/SecretomeP-1.0) and SignalP 6.0 (https://services.healthtech.dtu.dk/services/SignalP-6.0) online tools. If a (NN) score produced by SecretomeP 1.0f is higher than the threshold of 0.6, the protein is predicted as an unconventionally secreted protein. There were no N-terminal signal peptide sequences were predicted from EqPdt, EqIsc1 and MoCmu using the SignalP 6.0.

**Table. S2** The conservation of *E. quercicola* candidate effectors.

| Name | Gene ID | No. of other powdery mildew species that have homologs | | PAFM domain |
| --- | --- | --- | --- | --- |
| EqPdt | MK391403 | 8 | PDT, PF00800;  ACT, PF01842 | |
| EqCmu | MK391402 | 8 | CM_2, PF01817 | |
| EqIsc1 | QKX08450.1 | 4 | Isochorismatase, PF00857 | |
| EqCSEP0565 | PQ699781 | 7 | No | |
| EqCSEP0804 | PQ699782 | 3 | No | |
| EqCSEP0854 | PQ699783 | 9 | No | |
| EqCSEP1106 | PQ699784 | 5 | No | |
| EqCSEP1276 | MZ502496.1 | 2 | Collagen, PF01391 | |
| EqCSEP1329 | PQ699785 | 10 | No | |
| EqCSEP2231 | PQ699786 | 3 | No | |
| EqCSEP3479 | PQ699787 | 7 | No | |
| EqCSEP4187 | MZ365025.1 | 3 | No | |
| EqCSEP4728 | PQ699788 | 8 | No | |

**Table. S3** CMUs and PDTs in diverse organisms.

| Pathogenic  microorganisms | Organism | | Cmu | | Pdt | | Bifunctional  Cmu/Pdt | |
| --- | --- | --- | --- | --- | --- | --- | --- | --- |
|  |  |  | Gene ID | secretion | Gene ID | secretion | Gene ID | secretion |
|  | Fungi | *Sclerotinia sclerotiorum* | XP_001590829.1 | No | [APA07797.1](https://www.ncbi.nlm.nih.gov/protein/APA07797.1?report=genbank&log$=prottop&blast_rank=1&RID=N7GCTVWH013) | No |  | |
|  |  | *Bipolaris maydis* | XP_014080168.1 | Yes | [XP_014076484.1](https://www.ncbi.nlm.nih.gov/protein/XP_014076484.1?report=genbank&log$=prottop&blast_rank=1&RID=N7GD47NE016) | No |  | |
|  |  | *Botrytis cinerea* | KAK6600091.1 | No | XP_001555030.1 | Yes | EMR83441.1 | No |
|  |  | *Erysiphe quercicola* | MK391402 | Yes | MK391403 | Yes |  | |
|  |  | *Erysiphe necator* | KAI6247435.1 | Yes | KHJ34375.1 | Yes |  | |
|  |  | *Golovinomyces cichoracearum* | RKF58835.1 | Yes | [RKF74106.1](https://www.ncbi.nlm.nih.gov/protein/RKF74106.1?report=genbank&log$=prottop&blast_rank=1&RID=N7GN5Z5T016) | Yes |  | |
|  |  | *Blumeria graminis* | EPQ64889.1 | Yes | [EPQ63196.1](https://www.ncbi.nlm.nih.gov/protein/EPQ63196.1?report=genbank&log$=prottop&blast_rank=1&RID=N7GSTCV3016) | Yes |  | |
|  |  | *Blumeria hordei* | CCU75899.1 | Yes | CCU77384.1 | Yes |  | |
|  |  | *Pyricularia oryzae* | XP_003714705.1 | Yes | KAH8843245.1 | No |  | |
|  |  | *Ustilago maydis* | XM_011393174.1 (25) | Yes | XP_011386324.1 | No |  | |
|  | Oomycete | *Phytophthora sojae* | XP_009526259.1 | No | At2g22250(63) | No |  | |
|  | Bacteria | *Acidovorax citrulli* | UMT86803.1 | No | UMT88042.1 | No | ATG94418.1 (24) | No |
|  |  | *Xanthomonas oryzae* | EC5.4.99.5 (61) | Yes | UNE64219.1 | No |  | |
|  |  | *Xanthomonas arboricola* | AKJ12_RS15475 (58) | Yes | KOB34773.1 | No | WP_039520680.1 | No |
|  | Nematode | *Hirschmanniella oryzae* | KP297892  (57) | Yes |  | |  | |
|  |  | *Meloidogyne incognita* | AY422834.1 (59) | Yes |  | |  | |
| Non-pathogenic microorganisms | Fungi | *Saccharomyces cerevisia* | NP_015385.1 | No | QHB11078.1 | No |  | |
|  |  | *Schizosaccharomyces pombe* | NP_594216.1 | Yes | NP_001417966.1 | Yes |  | |
|  |  | *Purpureocillium lilacinum* | XP_065455783.1 | No | XP_018183825.1 | No |  | |
|  |  | *Monascus purpureus* | TQB68073.1 | No | TQB74184.1 | No |  | |
|  |  | *Aspergillus nidulans* | XP_664470.1 | No | AAY68231.1 | Yes |  | |
|  |  | *Neurospora crassa* | XP_961975 | No | KAK3504627.1 | No | XP_011393836.1 | No |
|  | Bacteria | *Escherichia coli* | KIH36130.1 | No | PPA51641.1 | No | BCA75054.1 | No |

**Note.** Secretory characteristics of these proteins were analyzed using SecretomeP 1.0f (http://www.cbs.dtu.dk/services/SecretomeP-1.0), SignalP 6.0 (https://services.healthtech.dtu.dk/services/SignalP-6.0) and SecretomeP - 2.0 (<https://services.healthtech.dtu.dk/services/SecretomeP-2.0/>) online tools.

**Table. S4** Primer information.

|  | Primer sequence (5’-3’) | |
| --- | --- | --- |
| qRT-PCR analysis for *EqCmu* gene | F | GAGGTGAGCAGGCAGAAA |
|  | R | GGTCATCAGATTCGCAAG |
| qRT-PCR analysis for *EqPdt* gene | F | ACTCAACCTGAAAAGCAT |
|  | R | ATCCCAACTACCTAAAAC |
| qRT-PCR analysis for *EqIcs1* gene | F | TCACCGAGAAGATTACAC |
|  | R | TAGCAGGAGTCCACATAC |
| qRT-PCR analysis for *EqTub2* gene | F | CAGATACTGTTGTGGAGCCC |
|  | R | TGACCTGGGAAACGAAGA |
| qRT-PCR analysis for *EqCSEP0565* gene | F | GGTGTTGGTGTAAGGGTG |
|  | R | TCTTCTGCGTTAGGAGTG |
| qRT-PCR analysis for *EqCSEP0804* gene | F | GGAGCTAGAGCCCGTATC |
|  | R | GACCGAAGGACTGGAGTT |
| qRT-PCR analysis for *EqCSEP0854* gene | F | ATACCCATAAATCCCACG |
|  | R | ACTTCTTCTGGCAATCGT |
| qRT-PCR analysis for *EqCSEP1106* gene | F | TAGCGTAGCAACAAACCC |
|  | R | CAACCCATACTTCGTCCT |
| qRT-PCR analysis for *EqCSEP1276* gene | F | GCTTTAGCCTTAGGAGTT |
|  | R | GTTTGAGTTTGGGATGTT |
| qRT-PCR analysis for *EqCSEP1329* gene | F | ATCCTGCCAACATTACCT |
|  | R | CAAGAACACCAACAACCC |
| qRT-PCR analysis for *EqCSEP2231* gene | F | ACCTCGACGACAACTCTT |
|  | R | GCTTTATTTCCCATCACAT |
| qRT-PCR analysis for *EqCSEP3479* gene | F | TTCGTAGCCGACAAGATG |
|  | R | ACAGATGGTGGTGCGTAG |
| qRT-PCR analysis for *EqCSEP4187* gene | F | CTACGAAAGAGGACCGAGTA |
|  | R | GGTTTTCTCCCGCAGCTCTT |
| qRT-PCR analysis for *EqCSEP4728* gene | F | TTAGCCTTTATGGAGAAC |
|  | R | GAACCCTTGGAGTAGTAGA |
| qRT-PCR analysis for *EqEF-1a* gene | F | GAACCTTCATCTAACTGC |
|  | R | GTCGTAGTGGTTTGTCAG |
| qRT-PCR analysis for *HbPR1* gene | F | TAGGCTTAGCGATGGTGC |
|  | R | CTCCCTTGCGTTTATTGG |
| qRT-PCR analysis for *HbNPR1* gene | F | GAAACCCTATCCACTCCC |
|  | R | AGTCCTTTGCCAACTCCT |
| qRT-PCR analysis for *HbICS1* gene | F | GTAGAAGCCGAAGAAAGG |
|  | R | AACAGCAGAGCCTACACC |
| qRT-PCR analysis for *HbActin* gene | F | CTGGTGTTATGGTTGGGATG |
|  | R | CTCGGTGAGAAGCACTGGA |
| dsRNA synthesis for *EqCmu* | F | TAATACGACTCACTATAGGGATGGATGCTGCAGTAGATCT |
|  | R | TAATACGACTCACTATAGGGTGTCATTTAAGTTAATATTA |
| dsRNA synthesis for *EqPdt* | F | TAATACGACTCACTATAGGGATGGCGTCTTTACAGTCATCC |
|  | R | TAATACGACTCACTATAGGGGTAAGGCTTCTAATGTATCT |
| dsRNA synthesis for *EqTub2* | F | TAATACGACTCACTATAGGGATGACCTGGCACTATGGACG |
|  | R | TAATACGACTCACTATAGGGGTGATGGAACAACCGAAA |
| dsRNA synthesis for *GFP* | F | TAATACGACTCACTATAGGGGTGAGCAAGGGCGAG |
|  | R | TAATACGACTCACTATAGGGTTGTACAGCTCGTCCAT |
| Construction for pBin4-*EqCmu*-GFP vector | F | CCCcccgggATGGATGCTGCAGTAGATCTCTTG |
|  | R | CGggatccATCTGATAAACGTTGCATCAGGTAAT |
| Construction for pGBKT7/pGADT7-*EqCmu* vector | F | CGgaattcATGGATGCTGCAGTAGATCTCTTG |
|  | R | CGggatccATCTGATAAACGTTGCATCAGGTAAT |
| Construction for pET28a-*EqCmu*-His vector | F | CGggatccATGGATGCTGCAGTAGATCTC |
|  | R | CCGctcgagATCTGATAAACGTTGCATCAG |
| Construction for pSPYCE-*EqCmu* vector | F | CGCggatccGCGATGGATGCTGCAGTA |
|  | R | CCGctcgagCGGATCTGATAAACGTTGCA |
| Construction for pCambia-*EqCmu*-Flag vector | F | CCCcccgggGGGATGGATGCTGCAGTAGAT |
|  | R | CGCggatccGCGATCTGATAAACGTTGCATCAGGT |
| Construction for pBin4-*EqPdt*-GFP vector | F | TCCCcccgggATGCCTGATATGCAGCTTATGC |
|  | R | CGggatccACGAATTGATTTTGGTTTTAATTGA |
| Construction for pCambia-*EqPdt*-Flag vector | F | CGGggtaccCCGATGCCTGATATGCAGCTTATG |
|  | R | CGCggatccGCGCGAATTGATTTTGGTTTTAAT |
| Construction for pGEX6p-*EqPdt*-GST vector | F | CGggatccATGCCTGATATGCAGCTTATGC |
|  | R | CCGctcgagCTAACGAATTGATTTTGGTTTTAATTG |
| Construction for pET28a-*EqPdt*-His vector | F | CGggatccATGCCTGATATGCAGCTTATG |
|  | R | CCGctcgagACGAATTGATTTTGGTTTTAA |
| Construction for pYES2-*EqPdt*-HA vector | F | CGggatccATGCCTGATATGCAGCTTATGC |
|  | R | CCGctcgagCTAAGCGTAGTCTGGGACGTCGTATGGGTA  ACGAATTGATTTTGGTTTTAATTG |
| Construction for pSUC2-*EqPdt*-vector | F | ggaattttaattaagaattcATGCCTGATATGCAGCTTATGC |
|  | R | actatagggagaacctcgagACGAATTGATTTTGGTTTTAATTG |
| Construction for pSUC2-*EqPdt*^N100^ vector | F | CGgaattcATGCCTGATATGCAGCTTATGC |
|  | R | CCGctcgagATTGAGGTAGGTTTCCTTACAAATATTG |
| Construction for pSUC2-*EqPdt*^C100^ vector | F | ggaattttaattaagaattcATGAAGAGTGGTCTGTCAAACAAGACAA |
|  | R | actatagggagaacctcgagACGAATTGATTTTGGTTTTAATTGA |
| Construction for pSUC2-*MoCmu* vector | F | CCGgaattcCGGATGGAATCTTTGATAG |
|  | R | CCGctcgagCGGGGCTGCCGGCTGCAGCCTT |
| Construction for pSUC2-*MoCmu*^N100^ vector | F | CCGgaattcCGGATGGAATCTTTGATAG |
|  | R | CCGctcgagCGGATCAGGTGGAACGTGA |
| Construction for pGBKT7/pGADT7-*EqPdt* vector | F | TCCCcccgggATGCCTGATATGCAGCTTATGC |
|  | R | CGggatccACGAATTGATTTTGGTTTTAATTGA |
| Construction for pYES2-*ScPha2*-HA vector | F | CGggatccATGGCCAGCAAGACTTTGAGG |
|  | R | CCGctcgagTTAAGCGTAGTCTGGGACGTCGTATGGGTATTTGTG  ATAATATCTCTCATTTCTGGG |
| Construction for pSUC2-*ScPha2* vector | F | CGgaattcATGGCCAGCAAGACTTTGAGG |
|  | R | CCGctcgagTTTGTGATAATATCTCTCATTTCTGGG |
| Construction for pSPYNE-*EqPdt* vector | F | CGCggatccGCGATGCCTGATATGCAGCTTAT |
|  | R | CCGctcgagCGGACGAATTGATTTTGGT |
| Construction for pCambia-*GUS*-Flag vector | F | CGGggtaccCCGATGTTACGTCCTGTAGAAACC |
|  | R | CGCggatccGCGAAAGCCGCCGACTT |
| Construction for pJNARG-*EqCmu*-GFP vector | F | TTTCGTAGGAACCCAATCTTCAAAgaattcATGGATGCTGCAGTAGATCTC |
|  | R | GAACCACGATTAAATCGAGCCATgaattcATCTGATAAACGTTGCATCA |
| Construction for pJNARG-*EqPdt*-GFP vector | F | CGTAGGAACCCAATCTTCAAAgaattcATGCCTGATATGCAGCTTATG |
|  | R | GAACAGCTCCTCGCCCTTGCTCACaagcttACGAATTGATTTTGGTTTTAA |
| Construction for pJNARG-*MoCmu*-GFP vector | F | GGAACCCAATCTTCAAAgaattcATGGAATCTTTGATAGACCTGTC |
|  | R | GATTAAATCGAGCCATgaattcGGCTGCCGGCTGCAGC |
| Construction for pXY202-*BAS1*-mCherry vector | F | tttcgtaggaacccaatcttcaaaCGTGCCTAGGCGACTGCCAACCGTGA |
|  | R | ttcgaatttagcagcagcggtttctttTTTGTAAAGCTCATCCATAC |
